# Estimating Country-Specific Incidence Rates of Rare Cancers: Comparative Performance Analysis of Modeling Approaches Using European Cancer Registry Data

**DOI:** 10.1093/aje/kwab262

**Published:** 2021-10-29

**Authors:** Diego Salmerón, Laura Botta, José Miguel Martínez, Annalisa Trama, Gemma Gatta, Josep M Borràs, Riccardo Capocaccia, Ramon Clèries, for the Information Network on Rare Cancers (RARECARENet) Working Group

**Keywords:** credible interval, Poisson regression, random effects, rare events, uniform prior

## Abstract

Estimating incidence of rare cancers is challenging for exceptionally rare entities and in small populations. In a previous study, investigators in the Information Network on Rare Cancers (RARECARENet) provided Bayesian estimates of expected numbers of rare cancers and 95% credible intervals for 27 European countries, using data collected by population-based cancer registries. In that study, slightly different results were found by implementing a Poisson model in integrated nested Laplace approximation/WinBUGS platforms. In this study, we assessed the performance of a Poisson modeling approach for estimating rare cancer incidence rates, oscillating around an overall European average and using small-count data in different scenarios/computational platforms. First, we compared the performance of frequentist, empirical Bayes, and Bayesian approaches for providing 95% confidence/credible intervals for the expected rates in each country. Second, we carried out an empirical study using 190 rare cancers to assess different lower/upper bounds of a uniform prior distribution for the standard deviation of the random effects. For obtaining a reliable measure of variability for country-specific incidence rates, our results suggest the suitability of using 1 as the lower bound for that prior distribution and selecting the random-effects model through an averaged indicator derived from 2 Bayesian model selection criteria: the deviance information criterion and the Watanabe-Akaike information criterion.

## Abbreviation

DIC: deviance information criterion
INLA: integrated nested Laplace approximation
IR: incidence rate
LB: lower bound
MCMC: Markov chain Monte Carlo
RARECARENet: Information Network on Rare Cancers
RC: rare cancers
RMSE: root mean squared error
UB: upper bound
WAIC: Watanabe-Akaike information criterion

***Editor’s note:** An invited commentary on this article appears on page 499, and the authors’ response appears on page 503.*

Estimation of epidemiologic indicators of incidence, survival, and prevalence for rare cancers (RCs) is challenging, particularly for exceptionally rare entities and in countries with small populations. Because of the small–case-count data, the pros and cons of directly providing estimates based on very unstable empirical data or derived from modeling approaches are unclear, inherently less intuitive, more complex, and to some extent dependent on subjective choices.

Investigators in the Information Network on Rare Cancers (RARECARENet) (1–3) calculated incidence, prevalence, and survival estimates for an operative list of 190 RC entities, defined as cancers with incidence rates (IRs) less than 6 cases per 100,000 person-years, for the period 2000–2007 (3)*.* Since many countries could only provide data for cancer cases in some regional areas, not at a national level, RARECARENet investigators estimated country-specific IRs (number of cases per 100,000 person-years) for RCs on the basis of cases recorded by 83 population-based cancer registries across 27 European countries (3)*.* More recently, in the context of the Joint Action on Rare Cancers, which is generating policy recommendations on RC that can be implemented by European Union member states (1–3), the burden of RC incidence counts in Europe was compared with another burden derived by a model-based approach that used a simple Poisson random-effects model under the Bayesian framework (4), through integrated nested Laplace approximations (INLAs) implemented in the INLA platform (5). Evidence suggests that INLA is appropriate for estimating the distribution of fixed-effect parameters, but it could fail to yield good estimates in a random-effects model (6). This last shortcoming could be related to the numerical method used in INLA to estimate the posterior distribution: Laplace approximation (4, 6). This approximation is good for models close to a Gaussian distribution, but this may underestimate the variance of the random effects when modeling Poisson or binary data (4, 6). In that situation, reasonable approximations to the posterior are achieved when modeling high counts (4) or by altering INLA’s default settings (6).

A previous study showed very small differences in the precision of certain indicators when implementing a simple Poisson random-effects model with different platforms and approaches (4); the authors called for a simulation study to properly analyze the differences in the context of RCs. These 190 entities represent different specific cancers, and their distribution may vary across countries; however, it is not feasible to use a different statistical model for each. We therefore aim to propose an approach that: 1) provides an overall better model fit for all of the entities; 2) shows the implication of using the Bayesian approach for deriving the corresponding 95% credible intervals; and 3) proposes prior distributions for the precision of random effects in specific situations related to small case counts.

## METHODS

We performed 2 studies. We designed study 1 as a simulation, comparing 4 RC scenarios to assess the performance of the frequentist, empirical Bayes, and Bayesian approaches in the presence of overdispersion. Study 2 assessed the choice of appropriate lower and upper bounds for a uniform prior distribution on the standard deviation, $\sigma$, of the random-effects model, depending on the IRs from 2000–2007. The accompanying Web material (available at https://doi.org/10.1093/aje/kwab262) provides technical details, R (version 3.5.1; R Foundation for Statistical Computing, Vienna, Austria) (7) and WinBUGS (MRC Biostatistics Unit, Cambridge, United Kingdom, and Imperial College School of Medicine, London, United Kingdom) (8) software code, and additional results.

### The Poisson random-effects model

In the 1980s, a relative risk regression model was proposed, assuming that the observed cases ${C}_i$ in the $i$th area (in our study country) were distributed according to a Poisson distribution (9), such that

(1) \begin{equation*} {C}_i\sim \mathrm{Poisson}\left({R}_i{E}_i\right),i=1,\dots, I=27, \end{equation*}

where ${R}_i$ is a relative risk or standardized incidence ratio and ${R}_i=E({C}_i)/{E}_i$, where $E({C}_i)={\lambda}_i$ and ${E}_i$ are, respectively, the expected rate for ${C}_i$ and the expected number of cases calculated from internal standardization or from an external source of age- and sex-specific rates. For each RC considered, the observed number of cases for the $i$th area/country covered by the cancer registries was modeled as in equation 1, but accounting for extra Poisson variability through a Poisson random-effects model presenting a hierarchical structure (9–15):

$$ {\theta}_i=\log \left({R}_i\right)=\mu +{\nu}_i,\ i=1,\dots, I=27, $$

(2) \begin{equation*} {\nu}_i\sim N\left(0,\tau \right), \end{equation*}

where the parameter $\mu$ is the unknown intercept and ${\nu}_i$ are the random effects representing the unstructured residual for each country, assuming a Gaussian distribution with mean 0 and precision $\tau$, whereas ${\theta}_i$ is the log relative risk, that is, ${R}_i={e}^{\theta_i}$. In this approach, also known as the exchangeable random intercept approach, the distribution of the random effects allows for extra-Poisson variability in the marginal distribution of the ${C}_i$’s.

Once the model parameters were estimated, the expectation of the predicted number of incident cases for the *k*th country was calculated through ${\hat{\lambda}}_k=\hat{R_k}{E}_k$, where $\hat{R_k}$ is an estimate of ${R}_k$.

### Study 1: simulation study

To evaluate the performance of the different approaches, we simulated data to establish a realistic ground truth for disease risk variation based on the validated and published data used in the RARECARENet project (1–4). Four cancer entities were selected from the RARECARENet database, and for each cancer entity, we simulated *J* = 1,000 data sets, those derived from fitting the original data set to model 2 (equation 2) through WinBUGS. Each simulated data set included *I* = 27 predicted values (one for each country) ${C}_i^j$, where *j* = 1, …, *J* and *i* = 1, …, *I*. We considered it realistic to include the 27 European Union member states in the study, since the aggregation of observed cases provided by the cancer registries within each country was previously validated to derive the most recent indicators: the country-specific number of RCs and their corresponding rates (1–4). In addition, in the context of RCs, an outcome of interest is to estimate the variability of the country-specific counts (4).

Table 1 shows the distribution of the original numbers of cases for the selected cancers: 2 with relatively large case counts (tumors of the central nervous system and adenocarcinoma with variants of ovary) and 2 with smaller counts (adenocarcinoma with variants of middle ear and adenocarcinoma with variants of trachea).

**Table 1:** Numbers of Observed Cases of 4 Selected Cancers Obtained From the RARECARENet Database^a^ During 2000–2007

	**No. of Observed Cases**		
**Cancer Entity**	**Range**	**Median**	**IR** ^**b**^
Tumors of the CNS^c^	174–28,732	2,758.0	73.800
AC with variants of ovary^c^	96–23,957	3,446.0	59.700
AC with variants of middle ear	0–17	1.8	0.003
AC with variants of trachea	0–39	6.0	0.011

Abbreviations: AC, adenocarcinoma; CNS, central nervous system; IR, incidence rate; RARECARENet, Information Network on Rare Cancers.

^a^ Numbers of cases were provided by 83 population-based cancer registries from 27 European countries (3).

^b^ Number of cases per 100,000 person-years, age-standardized to the European standard population (see Botta et al. (4)).

^c^ This cancer site was not considered a rare entity (large number of cases).

In order to simulate data sets similar to the original data, the estimates of the model 2 (equation 2) parameters were used as the true values. Specifically, for each entity, if ${\mu}_0$ and ${\tau}_0$ were the posterior estimates for $\mu$ and $\tau$ given the original data used in the RARECARENet project (obtained using the WinBUGS code of Web Appendix 1), where $\sigma \sim \mathrm{Uniform}(\mathrm{LB},\mathrm{UB})$ with lower bound (LB) = 0 and upper bound (UB) = 500 and ${\tau}_0=\frac{1}{({\sigma}^2)})$, then for $j=1,\dots, J=\mathrm{1,000}$, and for $i=1,\dots, I=27$, we simulated ${\nu}_i^j\sim N(0,{\tau}_0)$, we computed ${R}_i^j=\exp ({\mu}_0+{\nu}_i^j)$, and we generated the “new” observed cases ${C}_i^j\sim \mathrm{Poisson}({R}_i^j{E}_i)$. The simulated distribution of the “new” observed cases for each country is graphically depicted in Web Appendix 2 and Web Figures 1–4.

We compared the performance of 13 computational approaches (see Web Appendices 3–5) for estimating the mean number of cases and their confidence/credible intervals by using maximum likelihood approaches, empirical Bayes approaches, and modeling (12–22). The Bayesian models were fitted using WinBUGS and INLA (see Web Appendices 1, 4, and 5 for implementation and Web Appendices 3, 6, and 7 for details and computational issues (23–27)). There were 7 WinBUGS models fitted by assuming that σ follows a uniform prior with different LBs and UBs (see their code implementation in Web Appendix 1): In model 1, LB = 0 and UB = 500; in model 2, LB = 0.1 and UB = 500; in model 3, LB = 0.2 and UB = 500; in model 4, LB = 0.3 and UB = 500; in model 5, LB = 0.5 and UB = 500; in model 6, LB = 1 and UB = 500; and in model 7, there was sampling from LB and UB (see Web Appendix 5) such that LB $\sim\!\! \mathrm{Uniform}(0,5)$ and UB $\sim \mathrm{Uniform}(\mathrm{LB},500)$. INLA models were fitted by assuming 2 priors on the precision and standard deviation of the random effects: model 8, in which $\tau$ followed a γ prior with $\alpha =\beta =0.000001$ (the INLA model used by Botta et al. (4)) and model 9 (see Web Appendix 4), where σ had a uniform prior distribution between 0 and $\infty$.

These 9 models were also compared with another 4 approaches: model 10—an empirical Bayes approach assuming a gamma distribution (14–16) for ${R}_i^j$; model 11—a generalized linear mixed model estimated by maximum likelihood and 2 standard approaches not requiring modeling; model 12—exact Poisson distribution; and model 13—Byar’s Poisson approximation (11, 13). The last 2 approaches were used to compare the coverage and width of their 95% confidence intervals with the intervals obtained using modeling.

For each cancer entity and simulated data set, we calculated the 95% confidence interval or credible interval for the posterior number of cases in each country, ${\hat{\lambda}}_i^j={\hat{R}}_i^j{E}_i$, measuring 3 indicators: 1) width, 2) coverage, and 3) the root mean squared error (RMSE) between ${\lambda}_i$ and ${\hat{\lambda}}_i^j={\hat{R}}_i^j{E}_i$ (assuming that ${R}_i$ was the “real” value for the relative risk in the *i*th country, whereas ${\hat{R}}_i^j$ was its estimate).

Bayesian and non-Bayesian approaches were assessed in terms of confidence and credible intervals by comparing the performance of these intervals through the percentage of their respective coverage of the true ${\lambda}_i$ parameter across the 1,000 simulated data sets for each of the 4 cancer entities considered. However, one has to distinguish the interpretation of confidence and credible intervals, since they are conceptually different. The 95% confidence intervals refer to how often these intervals, computed from repetitions of the experiment under study, would contain the true parameter—considered as fixed—if model assumptions were valid (28). On the other hand, if one is interested in computing an interval with a 95% probability of containing a model parameter, considered as random, where each numerical value contained within this interval has its own probability mass, then the resulting interval is the Bayesian credible interval (18). For credible intervals, the achievement of the frequentist coverage when replicating a study is a desirable property for the prior’s assessment, in order to yield reliable posterior inference (29). It also guarantees a frequentist validity of these intervals when compared with confidence intervals (29). An adequate confidence/credible interval is expected to have coverage values of at least its nominal value, and if we calculate the average width of these intervals for all data sets, the one producing the narrowest width is preferred (29).

In addition to these 3 indicators, the effective number of model parameters (*pD*), the mean deviance, and the deviance information criterion (DIC) were also calculated (23) and compared between Bayesian models (see Web Appendices 6 and 7).

### Study 2: applied study

In study 2, we compared the performance of models 1–7 across the 190 cancer entities by assessing the bounds of the uniform prior distribution in WinBUGS. For each cancer entity, we calculated 1) the average width of the 95% credible intervals of the 27 posterior estimates of ${\lambda}_i$, 2) the expected value of the posterior σ, and 3 common measures used by data analysts for assessing model fit under the Bayesian framework: 3) the DIC and 2 versions of the Watanabe-Akaike information criterion (WAIC) (30–32), designated 4) ${\mathrm{WAIC}}_1$ and 5) ${\mathrm{WAIC}}_2$ (see Web Appendices 8–10).

We compared these indicators between models in each cancer entity as follows. First, we determined the minimum value of the average width, the posterior σ, ${\mathrm{WAIC}}_1$,${\mathrm{WAIC}}_2$, and DIC across models. Second, for each model, we calculated the difference between these 5 indicators and their corresponding minimum. We stored these 5 indicators in a matrix of 190 rows, one for each cancer site selected from the RARECARENet database, and 40 columns (5 indicators × 8 models considered). We summarized the results (median and 2.5th and 97.5th percentiles of each difference) for a specific selection of cancer entities in order to assess model performance.

The selection was based on the magnitude of the IRs (number of cases per 100,000 person-years), since these showed a minimum of 0.0004 and a maximum of 5.9692, with a median of 0.1100. We chose 4 scenarios: scenario A, where the IR was less than 0.03 (quartile 1 of the IRs (47/190 cancer entities)); scenario B, where the IR was less than 0.12 (up to quartile 2 of the IRs (97/190 entities)); scenario C, where the IR was less than 0.5 (up to quartile 3 of the IRs (143/190 entities)); and scenario D, where the IR was greater than or equal to 0.5 (quartile 4 of the IRs).

Furthermore, to suggest a strategy for different scenarios, we established a ranking for each model and cancer entity by calculating the indicators and then averaging the rankings of indicators for each model in the corresponding scenario (see Web Appendix 11, where Web Figure 5 depicts this procedure).

Finally, we made a graphical comparison between the best model derived from study 2 and model 8.

## RESULTS

### Study 1

Table 2 presents modeling indicators for cancer sites with large case counts. For tumors of the central nervous system, we noted that 1) the mean coverage for the generalized linear mixed model and the empirical Bayes strategies barely reached 95% and 2) the 2 nonmodeling strategies, exact and Byar’s Poisson approximation, yielded wide confidence intervals. This phenomenon was also detected for an entity with large variability and relatively large counts: adenocarcinoma with variants of ovary. The Bayesian models showed an average of almost 95% coverage, with similar widths and RMSEs.

**Table 2:** Mean Values (and Standard Deviations) of Indicators Considered for Assessing the Performance of Models Used to Estimate Incidence of 2 Types of Cancer With Large Case Counts, RARECARENet Database, 2000–2007^a^

**Cancer Type and Model Used**	**CI/CrI Coverage, %**	**CI/CrI Width, no. of cases**	**RMSE**
Tumors of the central nervous system			
WinBUGS models^b^			
Model 1	95.16 (21.88)	223.62 (128.03)	46.38 (48.69)
Model 2	94.16 (22.18)	223.56 (127.57)	46.36 (48.76)
Model 3	95.33 (21.10)	224.08 (127.33)	46.43 (48.90)
Model 4	95.26 (21.26)	224.67 (127.30)	46.51 (46.51)
Model 5	95.26 (21.26)	224.67 (127.30)	46.51 (49.04)
Model 6	95.11 (21.57)	224.99 (127.35)	46.64 (49.11)
Model 7	94.89 (22.03)	223.66 (127.85)	46.44 (48.92)
INLA models^c^			
Model 8	95.18 (22.18)	223.81 (127.93)	46.44 (48.83)
Model 9	95.18 (22.18)	223.79 (127.90)	46.44 (48.84)
Empirical Bayes approach	94.96 (21.88)	222.33 (128.90)	46.38 (48.68)
Generalized linear mixed model	94.67 (22.48)	223.51 (128.35)	46.39 (48.75)
Poisson approximation			
Exact	95.04 (21.73)	226.20 (127.26)	46.64 (49.08)
Byar’s	95.11 (21.57)	225.22 (127.35)	46.64 (49.08)
Adenocarcinoma with variants of ovary			
WinBUGS models			
Model 1	95.04 (21.73)	204.59 (119.07)	40.98 (44.08)
Model 2	95.19 (21.42)	204.55 (118.76)	40.99 (43.85)
Model 3	95.04 (21.73)	204.56 (118.77)	40.92 (43.97)
Model 4	95.11 (21.57)	204.91 (118.57)	40.91 (43.94)
Model 5	95.11 (21.57)	204.91 (118.57)	40.91 (43.94)
Model 6	95.56 (20.62)	205.59 (118.47)	40.99 (43.88)
Model 7	95.11 (21.75)	204.55 (118.81)	40.98 (43.82)
INLA models			
Model 8	95.03 (22.03)	204.70 (118.71)	40.94 (43.88)
Model 9	95.03 (22.03)	204.77 (118.76)	40.93 (43.86)
Empirical Bayes approach	94.67 (22.33)	203.45 (118.84)	40.99 (43.87)
Generalized linear mixed model	94.83 (22.03)	204.32 (118.79)	40.93 (43.82)
Poisson approximation			
Exact	95.26 (21.26)	206.43 (118.08)	40.98 (43.88)
Byar’s	95.48 (20.78)	205.50 (118.20)	40.98 (43.87)

Abbreviations: CI, confidence interval; CrI, credible interval; INLA, integrated nested Laplace approximation; RARECARENet, Information Network on Rare Cancers; RMSE, root mean squared error.

^a^ Coverage and width of the 95% CI or CrI and RMSE between the observed and predicted numbers of cases across 1,000 simulated data sets for 2 cancer sites with large case counts in all areas.

^b^ WinBUGS models: model 1, $\sigma\!\!\sim\!\!\ \mathrm{Uniform}(0,500)$; model 2, $\sigma\!\!\sim\!\! \mathrm{Uniform}(0.1,500)$; model 3, $\sigma\!\!\sim\!\! \mathrm{Uniform}(0.2,500)$; model 4, σ $\sim \mathrm{Uniform}(0.3,500)$; model 5, σ $\sim \mathrm{Uniform}(0.5,500)$; model 6, σ $\sim \mathrm{Uniform}(1,500)$; model 7, σ $\sim \mathrm{Uniform}(a,b)$.

^c^ INLA models: model 8, $\tau \sim \gamma (0.00001,0.00001)$; model 9, σ $\sim \mathrm{Uniform}(0,\infty)$.

The indicators for cancer entities with very small counts and a large number of zeros are presented in Table 3. The exact Poisson and Byar’s Poisson strategies produced large confidence intervals. WinBUGS and INLA strategies using a uniform prior on $\sigma$ showed good performance compared with the aforementioned strategies, with narrower intervals and RMSEs, and coverage above 96%. Among WinBUGS models, we noted that model 6 showed the largest width (3.61 cases) and RMSE (RMSE = 0.62) and model 1 the smallest values. On the other hand, model 8 (INLA) presented low mean coverage (79.56%), well below the expected 95%, indicating that this model would not be appropriate for adenocarcinoma with variants of middle ear. These conclusions related to coverage can also be applied to the generalized linear mixed and empirical Bayes models.

**Table 3:** Mean Values (and Standard Deviations) of Indicators Considered for Assessing the Performance of Models Used to Estimate Incidence of 2 Types of Cancer With Small Case Counts, RARECARENet Database, 2000–2007^a^

**Cancer Type and Model Used**	**CI/CrI Coverage, %**	**CI/CrI Width, no. of cases**	**RMSE**
Adenocarcinoma with variants of middle ear			
WinBUGS models^b^			
Model 1	96.04 (16.96)	2.54 (2.54)	0.43 (0.69)
Model 2	96.56 (15.45)	2.63 (2.57)	0.43 (0.69)
Model 3	96.22 (13.22)	2.74 (2.61)	0.43 (0.69)
Model 4	96.59 (11.78)	2.86 (2.67)	0.44 (0.70)
Model 5	96.59 (11.78)	2.86 (2.67)	0.44 (0.70)
Model 6	96.37 (12.67)	3.61 (2.94)	0.62 (0.84)
Model 7	96.37 (12.67)	2.84 (2.65)	0.44 (0.70)
INLA models^c^			
Model 8	79.56 (40.34)	1.78 (2.23)	0.46 (0.76)
Model 9	95.59 (18.15)	2.57 (2.56)	0.44 (0.70)
Empirical Bayes approach	51.33 (50.00)	1.23 (2.12)	0.45 (0.70)
Generalized linear mixed model	76.44 (42.45)	1.84 (2.33)	0.45 (0.71)
Poisson approximation			
Exact	96.74 (17.76)	6.11 (2.87)	0.91 (0.99)
Byar’s	98.44 (12.38)	5.05 (2.94)	0.91 (0.99)
Adenocarcinoma with variants of trachea			
WinBUGS models			
Model 1	92.44 (26.44)	6.28 (5.40)	1.39 (1.85)
Model 2	92.81 (25.83)	6.34 (5.40)	1.38 (1.83)
Model 3	94.00 (23.76)	6.42 (5.39)	1.37 (1.82)
Model 4	95.48 (20.78)	6.57 (5.43)	1.36 (1.82)
Model 5	95.48 (20.78)	6.57 (5.43)	1.36 (1.82)
Model 6	96.22 (19.07)	7.60 (5.61)	1.55 (1.90)
Model 7	95.30 (23.20)	6.49 (5.42)	1.38 (1.83)
INLA models			
Model 8	87.04 (33.60)	5.92 (5.37)	1.44 (1.91)
Model 9	92.44 (26.44)	6.36 (5.41)	1.39 (1.83)
Empirical Bayes approach	84.15 (36.54)	5.58 (5.18)	1.39 (1.87)
Generalized linear mixed model	85.70 (35.02)	6.01 (5.38)	1.39 (1.84)
Poisson approximation			
Exact	95.19 (21.42)	9.68 (5.48)	1.78 (1.94)
Byar’s	97.70 (14.98)	8.67 (5.51)	1.78 (1.94)

Abbreviations: CI, confidence interval; CrI, credible interval; INLA, integrated nested Laplace approximation; RARECARENet, Information Network on Rare Cancers; RMSE, root mean squared error.

^a^ Coverage and width of the 95% CI or CrI and RMSE between the observed and predicted numbers of cases across the 1,000 simulated data sets for 2 cancer sites with small case counts in all areas.

^b^ WinBUGS models: model 1, $\sigma\!\!\sim\!\! \mathrm{Uniform}(0,500)$; model 2, $\sigma\!\!\sim\!\! \mathrm{Uniform}(0.1,500)$; model 3, $\sigma\!\!\sim\!\!\mathrm{Uniform}(0.2,500)$; model 4, σ $\sim \mathrm{Uniform}(0.3,500)$; model 5, σ $\sim \mathrm{Uniform}(0.5,500)$; model 6, σ $\sim \mathrm{Uniform}(1,500)$; model 7, σ $\sim \mathrm{Uniform}(a,b)$.

^c^ INLA models: model 8, $\tau \sim \gamma (0.00001,0.00001)$; model 9, σ $\sim \mathrm{Uniform}(0,\infty)$.

Similarly, for adenocarcinoma with variants of trachea, a cancer entity with a median of 6 cases per country during the study period and just 3 countries with 0 counts, the coverage was below 95% in all modeling strategies except for models 4–7 (WinBUGS). Among these models, model 6 again showed the largest width (7.60 cases) and RMSE (RMSE = 1.55), model 7 the smallest width, and models 4 and 5 the smallest RMSEs. Therefore, changes in the lower bound on the uniform prior assumed for σ might improve model performance. Differences between WinBUGS and INLA models were only detected for *pD* values (see Web Figure 6).

### Study 2

**Figure 1:**
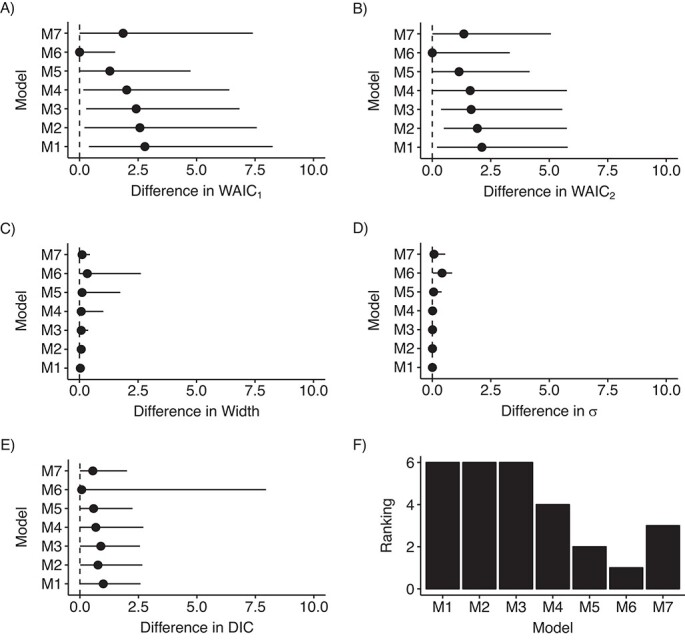
Assessment of models’ indicators (differences) and model (M) ranking in scenario A, where the incidence rate (IR) (number of cases per 100,000 person-years) was less than 0.03 (quartile 1 of the IRs (47/190 cancer entities)), RARECARENet database, 2000–2007. The graphs show median values (dots) and 95% credible intervals (lines) for the difference between the estimate of the corresponding indicator according to the specific model and its minimum across all models. A) Difference between the first version of the Watanabe-Akaike information criterion (WAIC_1_) for the corresponding model and the minimum WAIC_1_ across all models; B) difference between the second version of the Watanabe-Akaike information criterion (WAIC_2_) for the corresponding model and the minimum WAIC_2_ across all models; C) difference between the width of the 95% credible interval for ${\lambda}_i$ derived from the corresponding model and the minimum width of that interval across all models; D) difference between $\sigma$ estimated using the corresponding model and the minimum $\sigma$ estimated across all models; E) difference between the deviance information criterion (DIC) for the corresponding model and the minimum DIC across all models; F) ranking of the models according to the average of the rankings derived from the aforementioned indicators. See text for detailed descriptions of models 1–7. RARECARENet, Information Network on Rare Cancers.

**Figure 2:**
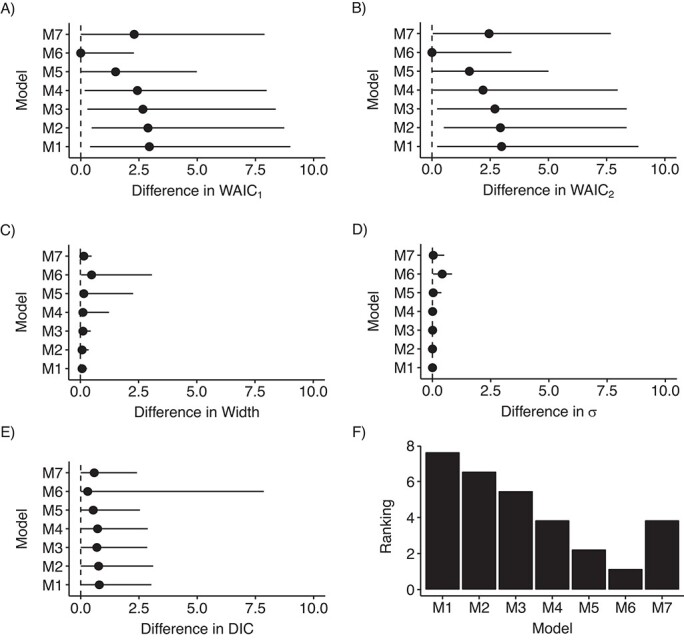
Assessment of models’ indicators (differences) and model (M) ranking in scenario B, where the incidence rate (IR) (number of cases per 100,000 person-years) was less than 0.12 (up to quartile 2 of the IRs (97/190 cancer entities)), RARECARENet database, 2000–2007. The graphs show median values (dots) and 95% credible intervals (lines) for the difference between the estimate of the corresponding indicator according to the specific model and its minimum across all models. A) Difference between the first version of the Watanabe-Akaike information criterion (WAIC_1_) for the corresponding model and the minimum WAIC_1_ across all models; B) difference between the second version of the Watanabe-Akaike information criterion (WAIC_2_) for the corresponding model and the minimum WAIC_2_ across all models; C) difference between the width of the 95% credible interval for ${\lambda}_i$ derived from the corresponding model and the minimum width of that interval across all models; D) difference between $\sigma$ estimated using the corresponding model and the minimum $\sigma$ estimated across all models; E) difference between the deviance information criterion (DIC) for the corresponding model and the minimum DIC across all models; F) ranking of the models according to the average of the rankings derived from the aforementioned indicators. See text for detailed descriptions of models 1–7. RARECARENet, Information Network on Rare Cancers.

**Figure 3:**
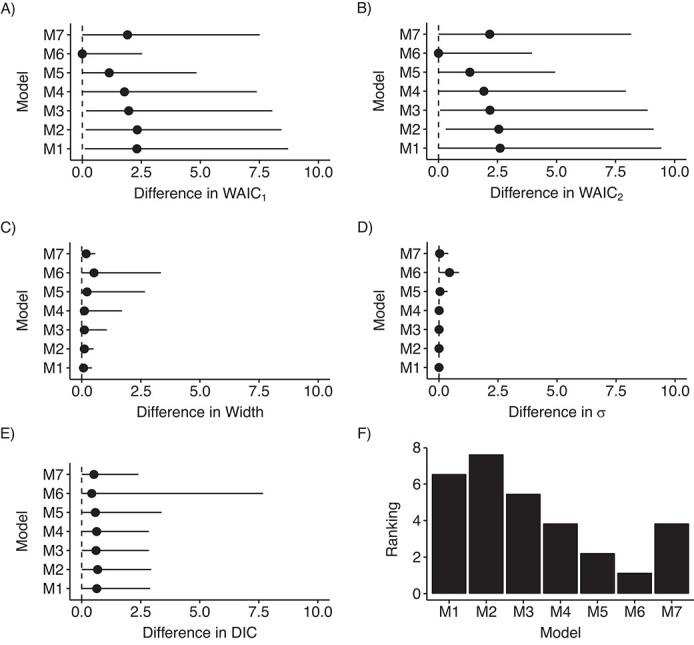
Assessment of models’ indicators (differences) and model (M) ranking in scenario C, where the incidence rate (IR) (number of cases per 100,000 person-years) was less than 0.5 (up to quartile 3 of the IRs (143/190 cancer entities)), RARECARENet database, 2000–2007. The graphs show median values (dots) and 95% credible intervals (lines) for the difference between the estimate of the corresponding indicator according to the specific model and its minimum across all models. A) Difference between the first version of the Watanabe-Akaike information criterion (WAIC_1_) for the corresponding model and the minimum WAIC_1_ across all models; B) difference between the second version of the Watanabe-Akaike information criterion (WAIC_2_) for the corresponding model and the minimum WAIC_2_ across all models; C) difference between the width of the 95% credible interval for ${\lambda}_i$ derived from the corresponding model and the minimum width of that interval across all models; D) difference between $\sigma$ estimated using the corresponding model and the minimum $\sigma$ estimated across all models; E) difference between the deviance information criterion (DIC) for the corresponding model and the minimum DIC across all models; F) ranking of the models according to the average of the rankings derived from the aforementioned indicators. See text for detailed descriptions of models 1–7. RARECARENet, Information Network on Rare Cancers.

**Figure 4:**
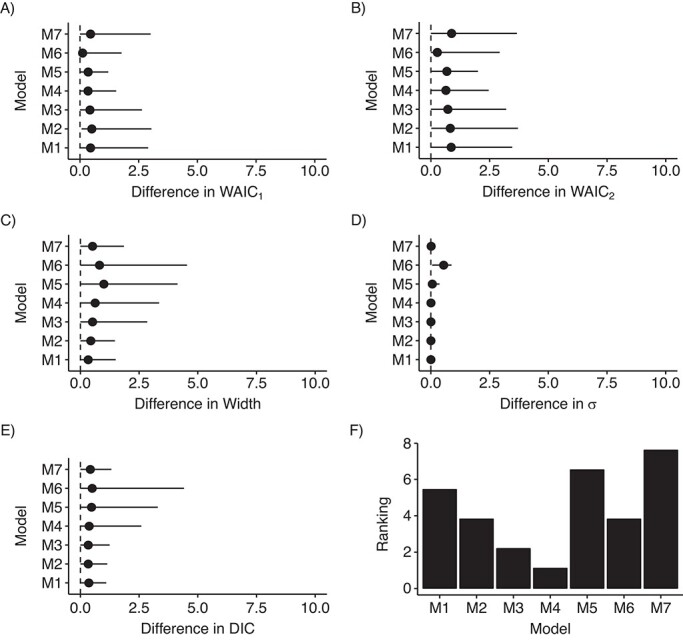
Assessment of models’ indicators (differences) and model (M) ranking in scenario D, where the incidence rate (IR) (number of cases per 100,000 person-years) was greater than or equal to 0.5 (quartile 4 of the IRs), RARECARENet database, 2000–2007. The graphs show median values (dots) and 95% credible intervals (lines) for the difference between the estimate of the corresponding indicator according to the specific model and its minimum across all models. A) Difference between the first version of the Watanabe-Akaike information criterion (WAIC_1_) for the corresponding model and the minimum WAIC_1_ across all models; B) difference between the second version of the Watanabe-Akaike information criterion (WAIC_2_) for the corresponding model and the minimum WAIC_2_ across all models; C) difference between the width of the 95% credible interval for ${\lambda}_i$ derived from the corresponding model and the minimum width of that interval across all models; D) difference between $\sigma$ estimated using the corresponding model and the minimum $\sigma$ estimated across all models; E) difference between the DIC of the corresponding model and the minimum DIC across all models; F) ranking of the models according to the average of the rankings derived from the aforementioned indicators. See text for detailed descriptions of models 1–7. RARECARENet, Information Network on Rare Cancers.

Since the results derived from the simulation study suggested that using a uniform prior distribution on $\sigma$ might be a useful strategy, we assessed the performance of models with different LBs on the uniform prior distribution for models 1–7. Figures 1–4 depict the distribution of these indicators across all scenarios. Model 6 showed the smallest differences on average, but it presented the largest variability for this last indicator. Model 6 ranked first among RC sites that had an IR of less than 0.5 cases per 100,000 person-years during the study period (Figure 1F, 2F, and 3F), whereas model 6 was surpassed by models 3 and 4 when the IR exceeded 0.5 (Figure 4F). Notably, model 6 performed best when DIC and WAIC rankings were averaged into one “overall indicator” (see Web Figure 7).

Finally, we also assessed the impact of using model 6 versus model 8 when comparing the 95% credible intervals for ${\lambda}_i$. For adenocarcinoma with variants of middle ear, the coverage of ${\lambda}_i$ using model 6 was clearly better than that with model 8, since the credible interval derived from model 8 did not cover the observed rate provided by RARECARENet in 5 countries (Web Figure 8). However, we noted that differences between model 6 and other models could also be minimal even for entities with very small case counts, such as adenocarcinoma with variants of trachea (Web Figure 9).

## DISCUSSION

In the Bayesian modeling of the burden of RC between countries, our study showed that using a uniform prior for the standard deviation of the random effects, $\sigma$, with a lower bound of 1, improves the performance of a simple Poisson random-effects model. However, when dealing with very large counts, the influence of the choice of the prior distribution or the non-Bayesian modeling might have a very small influence on the final results, as shown in study 1. In addition, model selection could be improved by selecting the model with the smallest ranking indicator derived from an average of the models’ rankings using DIC and WAIC. These 2 strategies are effective for modeling and obtaining a reliable measure of variability for country-specific RC IRs.

In the first study, we compared the coverage of the true ${\lambda}_i$ between Bayesian and non-Bayesian approaches. A common means of evaluating an objective prior distribution is through the frequentist-matching approach: If posterior credible intervals have good coverage properties, posterior inference is reliable in the absence of past data or sources of information (29). On the other hand, the bounds of the confidence interval and those of the credible interval for a parameter θ might numerically coincide when using a flat prior, *P*(θ) ∝ 1, since *P*(θ│*X*) ∝ *P*(θ)*L*(*X*│θ)—the posterior probability for θ, *P*(θ│*X*)—coincides with the likelihood (33), *L*(*X*│θ). However, use of the Bayesian approach allows the researcher to calculate probabilities for assessing whether certain parameter values are more probable than others.

Despite this fact, the Bayesian approach also has costs: 1) It adds dependency to the results due to the choice of prior distributions, and 2) it incurs a computational burden when using Markov chain Monte Carlo (MCMC) methods (6, 24, 34). The INLA platform performs approximate Bayesian inference based on the multiple use of Laplace approximations combined with numerical integration, providing faster computation than MCMC methods (6, 24, 25, 34–38), making its use appropriate for simple models. However, a limitation of INLA is related to the use of a bounded uniform prior distribution, since the end user cannot modify the bounds of a uniform prior (24, 25) as in WinBUGS (6, 24, 25, 34).

In the applied study, we used several indicators to assess the performance of the WinBUGS model for different LBs on the uniform prior distribution of $\sigma$. We gave the same weight to each of these indicators, and we ranked the models accordingly. However, we believe that the use of all of these indicators might be complex for applied data analysts. Since the most commonly used indicators for Bayesian model choice in epidemiologic studies are DIC and WAIC (30–32), our results suggest that averaging the models’ rankings using these indicators could be a good strategy. The DIC can easily be obtained from WinBUGS and INLA, whereas one can use the R code in Web Appendix 10 to carry out this calculation for WAIC indicators. In our study, we reached the same conclusions using these indicators as we did when using all indicators, except in the situation of relatively “large” IRs (IR > 0.5). We found that the best-performing model was that using $\sigma \sim \mathrm{Uniform}$(1, 500), so we suggest model 6 as a model to start with. However, there might be models that could perform better than model 6 for particular cancer entities (see Web Figure 10).

The relevance of choosing the prior distribution from an empirical Bayes perspective has been also reported (35) and mostly noted as a key factor from the full Bayesian perspective, where the comparative use of different priors is usually considered (19, 21, 22, 25, 34, 36). When estimating the rate of the RC counts through modeling the ratio between the observed and expected numbers of cases in each country, our results are a compelling argument for using a uniform prior distribution for $\sigma$ on the random effects. The end users can also assess the impact in the estimation and the variability of the model parameters by changing the LB of the uniform prior when using their own data.

Computational burden and differences in estimates could be an issue when comparing Bayesian platforms for modeling. INLA is much faster than platforms using MCMC methods, and this could be a key determinant of its use, since shorter computational time is a major advantage when analyzing large data sets. If computational time is not an issue, the flexibility in modeling priors through WinBUGS/MCMC is worth considering and can yield more accurate estimates of predictive probabilities than INLA. If computational time is an issue, we suggest using INLA and assessing a sensitivity analysis on the prior of $\tau$ by using γ-based priors (6, 14, 24, 25, 39–42). The use of INLA and combinations of MCMC and INLA have been suggested in situations dealing with hard-to-estimate conditional model parameters (6, 42, 43), such as in the case of geostatistical or spatiotemporal models (42). Here, using a simple random-effects model and *n* = 27 observations, all computations were carried out on a 4.8-GHz Intel Core i7 desktop personal computer (Intel Corporation, Santa Clara, California) with 16 GB of random access memory. The median computational time using model 6, fitted across the 190 cancer sites, was 227 minutes, whereas model 8 took 98 minutes.

### Further work

An overall European average for IRs was considered here, but spatial correlation between countries was not. In a scenario where yearly counts are available assuming between-area correlation, a model with identifiability constraints can help capture true spatial effects, and a Bayesian space-time model could also be used for modeling space × time interaction terms (42–44).

In this line, within-region variability is an important issue, especially for population-based cancer registry data. However, when dealing with RCs, the small counts often do not allow an in-depth study of the variability within a region from a statistical point of view, unless data are collected over a very long period of time (4). In addition, it is quite plausible that the within-region variability, if any, cannot be estimated with precision from a statistical viewpoint because of small counts. If our data were available disaggregated by local cancer registries within each country, model 2 (equation 2) could be easily modified to account for within-country variability by ${\theta}_{ir}=\log {R}_{ir}=\mu +{\nu}_i+{\alpha}_{ir}$. Here ${\nu}_i$ and ${\alpha}_{ir}$ are random effects representing the between-country and within-country variability, respectively, where ${\nu}_i\sim N(0,\tau)$, ${\alpha}_{ir}\sim N(0,{\tau}_{\alpha})$, *i* refers to country, and *r* refers to a within-country unit (local cancer registries). Although the disaggregation of data was not available at this level in the RARECARENet search tool (1–4) and that model cannot be tested with our data set, we suggest that this additional level of variability requires studying assumptions about the prior distributions for $\tau$ and ${\tau}_{\alpha }$ and how these can be combined and validated.

These assumptions must also take into account hypotheses—for example, that incidence is driven by an environmental risk factor present in one area but not another within the same country. In addition, population size has a significant influence on the measure of risk used in spatial modeling (44). These are challenges when applying a “one size fits all” method, and their future study in the context of RC is warranted.

### Limitations

The Joint Action on Rare Cancers strongly supported national/international coordination of clinical management of rare tumors, in terms of networking and physical centralization of treatments (1, 2). Therefore, provision of indicators at a subnational (local/regional cancer registry) level is of minor importance for health-care planning under the project’s aims. However, it remains an issue for etiological research on rare tumors with putative risk factors.

We studied the impact on the prior distributions used for the random effects, representing the unstructured residual for each country. The modeling and priors we used are only as reliable and valid as the data themselves (4). However, the modeling presented here might require additional assumptions in areas with poor cancer surveillance, where low occurrence might reflect issues with disease registration.

The database used here (March 10, 2020) was, unfortunately, the most updated one available at the time of this writing. This is largely due to the application of the European General Data Protection Regulation (45), which required a separate negotiation for data transfer with each one of the more than 100 participating cancer registries, so the speed of the entire data collection was determined by the slowest-reacting registry. Finally, the coronavirus disease 2019 pandemic emergency has entailed further delays over the entire process. We hope that the new database derived from the most recent “call for data” will become available during 2021, since it requires time for centralized processing and data quality checks.

### Conclusion

In summary, our study shows that a simple Bayesian Poisson regression model using a uniform prior distribution on $\sigma$ of the random effects with a lower bound of 1 yields reliable variability for the country-specific RC IRs when these vary around an overall IR. Despite this recommendation, in the context of RCs and small case counts, it is of the utmost importance to perform a sensitivity analysis combining precision with goodness of fit when the end user analyzes his/her own data. Along this line, we suggest selecting the random-effects model for each cancer site according to an averaged ranking indicator which uses DIC and WAIC.

## Supplementary Material

Web_Material_kwab262
