# Supplementary material for: Estimating Country-Specific Incidence Rates of Rare Cancers: Comparative Performance Analysis of Modeling Approaches Using European Cancer Registry Data
Source: Am J Epidemiol. 2021 Oct 29;191(3):487–98. doi: 10.1093/aje/kwab262 (PMC8895392; doi:10.1093/aje/kwab262)
Supplement: Web_Material_kwab262 [file web_material_kwab262.pdf]

WEB MATERIAL

Estimating Country-Specific Incidence Rates of Rare Cancers: Comparative Performance Analysis of Modeling Approaches Using European Cancer Registry Data

Contents

Web Appendix 1. WinBUGS Code: Poisson random-effects model..2

Web Appendix 2. Simulated distribution of the number of cases for the 4 entities considered in Study 1. 3

Web Figure 1...4

Web Figure 2...5

Web Figure 3...6

Web Figure 4...7

Web Appendix 3. Procedures and models used in the simulation study..8

Web Appendix 4. INLA code assuming σ~Uniform(0,∞)...11

Web Appendix 5. WinBUGS Code: Poisson random-effects model sampling LB and UB…..…. ….…12

Web Appendix 6. Measures for the assessment of the models/methods in the simulation study ..……..13

Web Appendix 7. Computational issues in the simulation study ……………………………….…15

Web Appendix 8. WAIC and log predictive pointwise density (LPPD) estimation using Markov chain Monte Carlo (MCMC)……………………………………………………………………………16

Web Appendix 9. A note on model selection methods used in this paper ……………………….....17

Web Appendix 10. R Code: WAIC calculations from WinBUGS output…………………………......18

Web Appendix 11. Illustration on the calculation of model's ranking…………………………………..19

Web Figure 5. Steps to rank models for each entity ………………………………………………..19

Web Figure 6. Differences in DIC and pD between INLA models M8 and M9 ………………………..20

Web Figure 7. Ranking of models accounting for DIC, WAIC1, WAIC2……………………………….…21

Web Figure 8. Comparison of performance between M6 and M8 for adenocarcinoma with variants of middle ear……………………………………………………………………………………...….22

Web Figure 9. Comparison of performance between M6 and M8 for adenocarcinoma with variants of trachea…………………………………………………………………………………………….23

Web Figure 10. Ranking of models accorss the 190 cancer entities…………………………………….….24

Web References…………………………………………………………………………………...……25

WEB APPENDIX 1

WinBUGS Code: Poisson Random-Effects Model

```
for (i in 1:n.countries)
{
		C[i] ~ dpois(lambda[i])
		log(lambda[i]) <- log(E[i]) + mu + nu[i]
		nu[i] ~ dnorm(0, tau) # area random effects
		theta[i] <- mu + nu[i] # area log-relative risk
		R_hat[i]<-exp(theta[i])
		# Posterior expectancy of Cases based on the R[i] posterior
		C_hat[i]<-R_hat[i]*E[i]
}

mu ~ dflat() # uniform prior on overall intercept
sigma ~ dunif(LB, UB) # sd area-random effects
tau <- 1/pow(sigma,2) # between-area precision


# Assume that this model is in file Model1.txt, where LB=0, UB=1

WinBUGS.model <-
bugs(data,inits,model.file='C:/Study/Model1.txt',parameters=c('lambda','tau','alpha'),n.
chains=3,n.iter=20000,n.burnin=10000,n.thin=10,bugs.directory='c:/WB',codaPkg=FALSE,debu
g=F)
```

WEB APPENDIX 2

Simulated Distribution of the Number of Cases for the 4 Entities Considered in Study 1

To evaluate the performance of the different approaches, we simulated data to establish a realistic ground truth for disease risk variation, based on the validated and published data used in RARECARENet project. Four cancer entities were selected from the RARECARENet database and each was fitted to a random-effects Bayesian model. Using the predictive distribution derived from this model, J=1000 simulations of a set of I=27 predicted values (one for each country) were performed, C_i^j, where j=1,…,J and i=1,…,I. We considered this realistic since the observed cases for the 27 European countries included in the study were previously validated to derive the most recent indicators: the country-specific number of RCs and their corresponding rates. In addition, in the context of rare cancers, an outcome of interest is to estimate the variability of the case counts.

In order to simulate data sets similar to the original data, we used the estimates of the model parameters as the true values, and then we simulated the model described in equation [2] of the main text, assuming overdispersion. Specifically, for each entity, if μ_0 and τ_0 were the posterior estimates for μ and τ given the original data used in the RARECAREnet project, (obtained using the WinBUGS code of Web Appendix 1, where $\sigma \sim Uniform(LB, UB)$ with lower bound (LB)=0 and upper bound (UB)=500 and $\tau_0=\frac{1}{(\sigma^2)}$), then for $j = \{1, \dots, J = 1000\}$, and for $i = \{1, \dots, I = 27\}$, we simulated $\nu_i^j \sim N(0, \tau_0)$, we computed $R_i^j = \exp(\mu_0 + \nu_i^j)$, and we generated the 'new' observed cases $C_i^j \sim Poisson(R_i^j E_i)$. The simulated distribution of the new observed cases derived from this model and for each cancer entity is depicted in Web Figures 1-4. There are two with "large" counts, tumours of the central nervous system (Web Figure 1) and adenocarcinoma with variants of ovary (Web Figure 3), and another two with "small" counts, adenocarcinoma with variants of middle ear (Web Figure 2) and adenocarcinoma with variants of trachea (Web Figure 4).

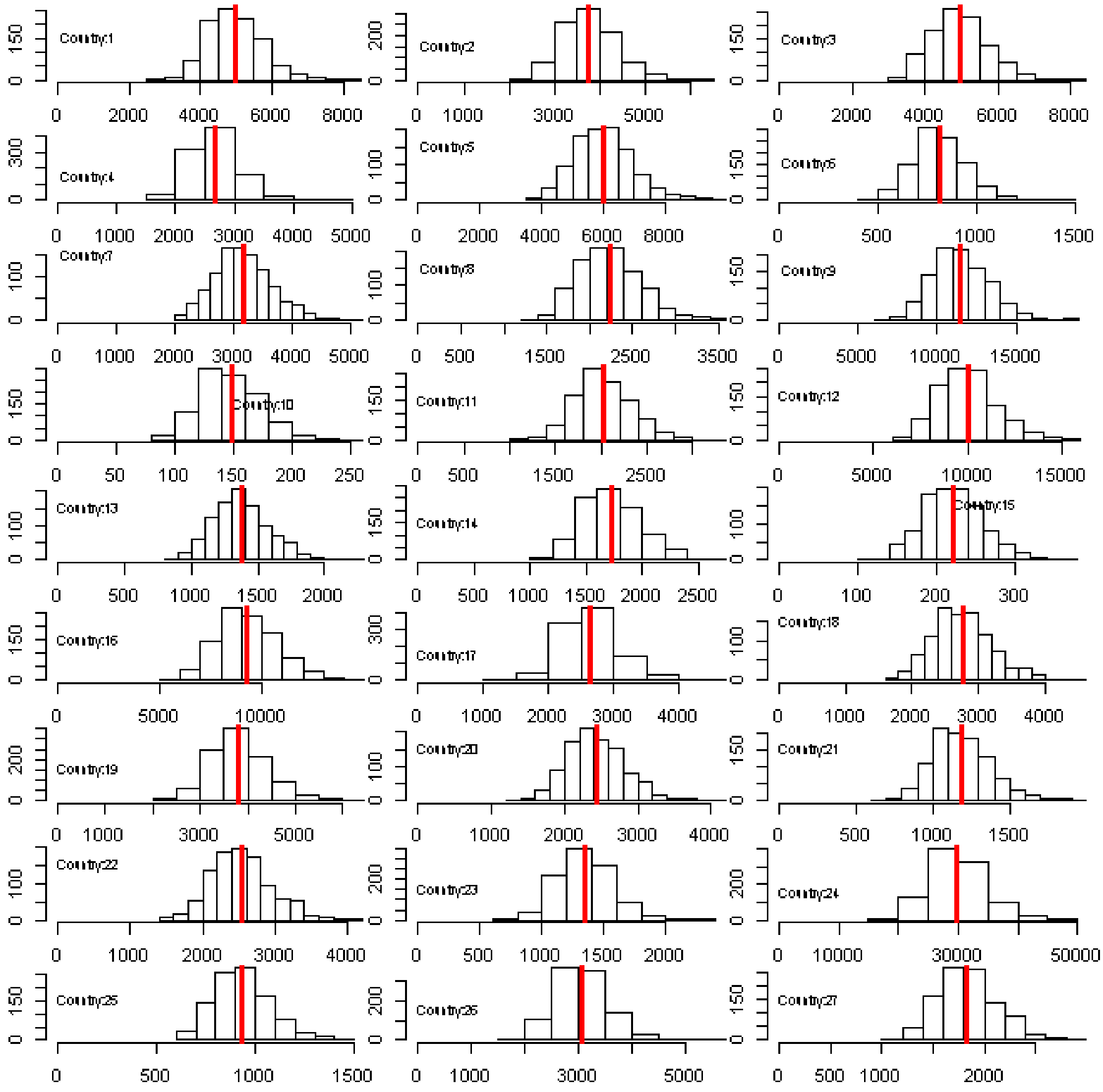

Web Figure 1. Distribution of the simulated cases (histogram based on C_i^j j=1,…,1000) for entity 1 (in red: actual observed cases in the corresponding country, that is C_i) for each of the 27 countries considered.

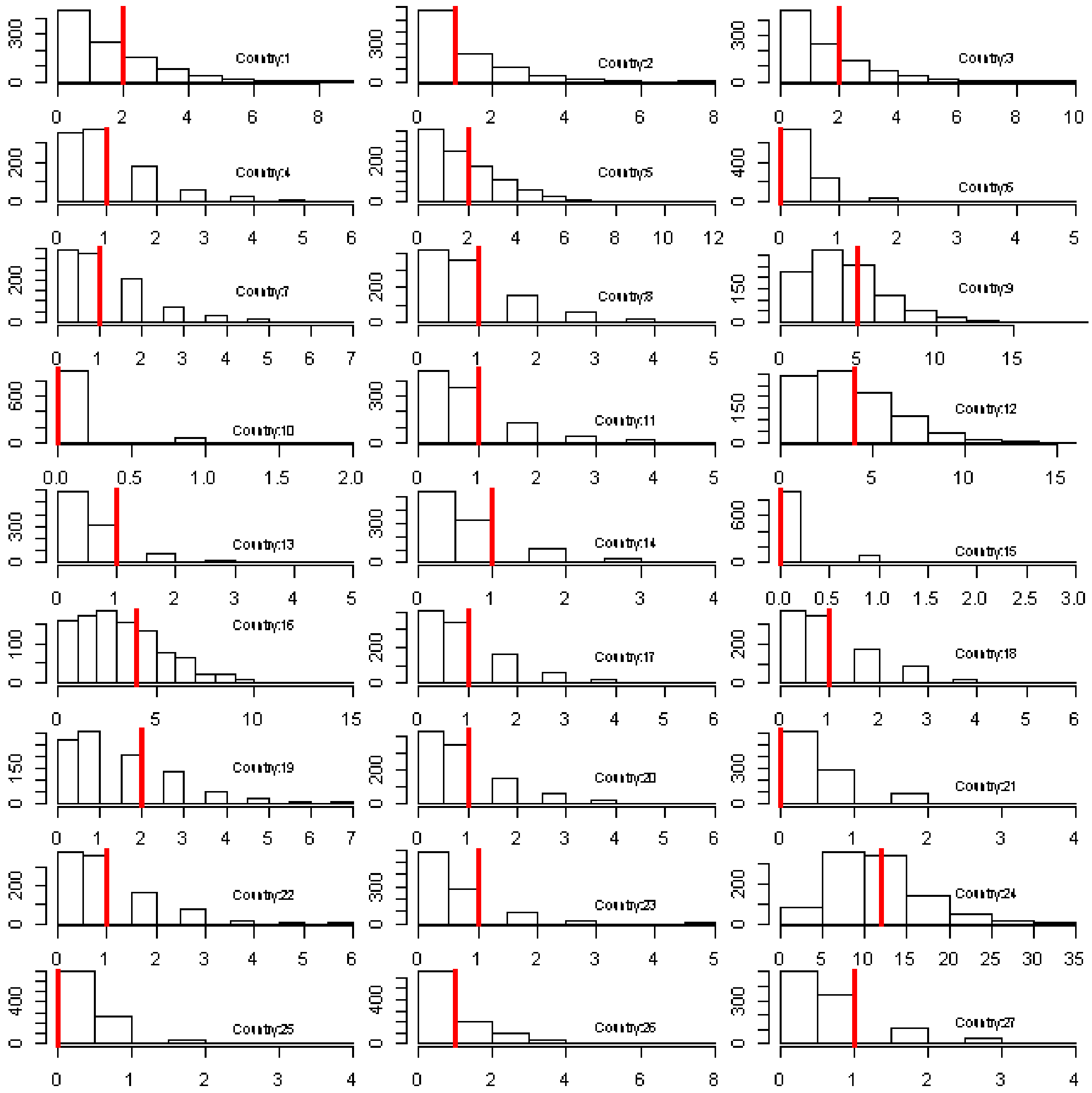

Web Figure 2. Distribution of the simulated cases (histogram based on C_i^j j=1,…,1000) for entity 2 (in red: actual observed cases in the corresponding country, that is C_i) for each of the 27 countries considered.

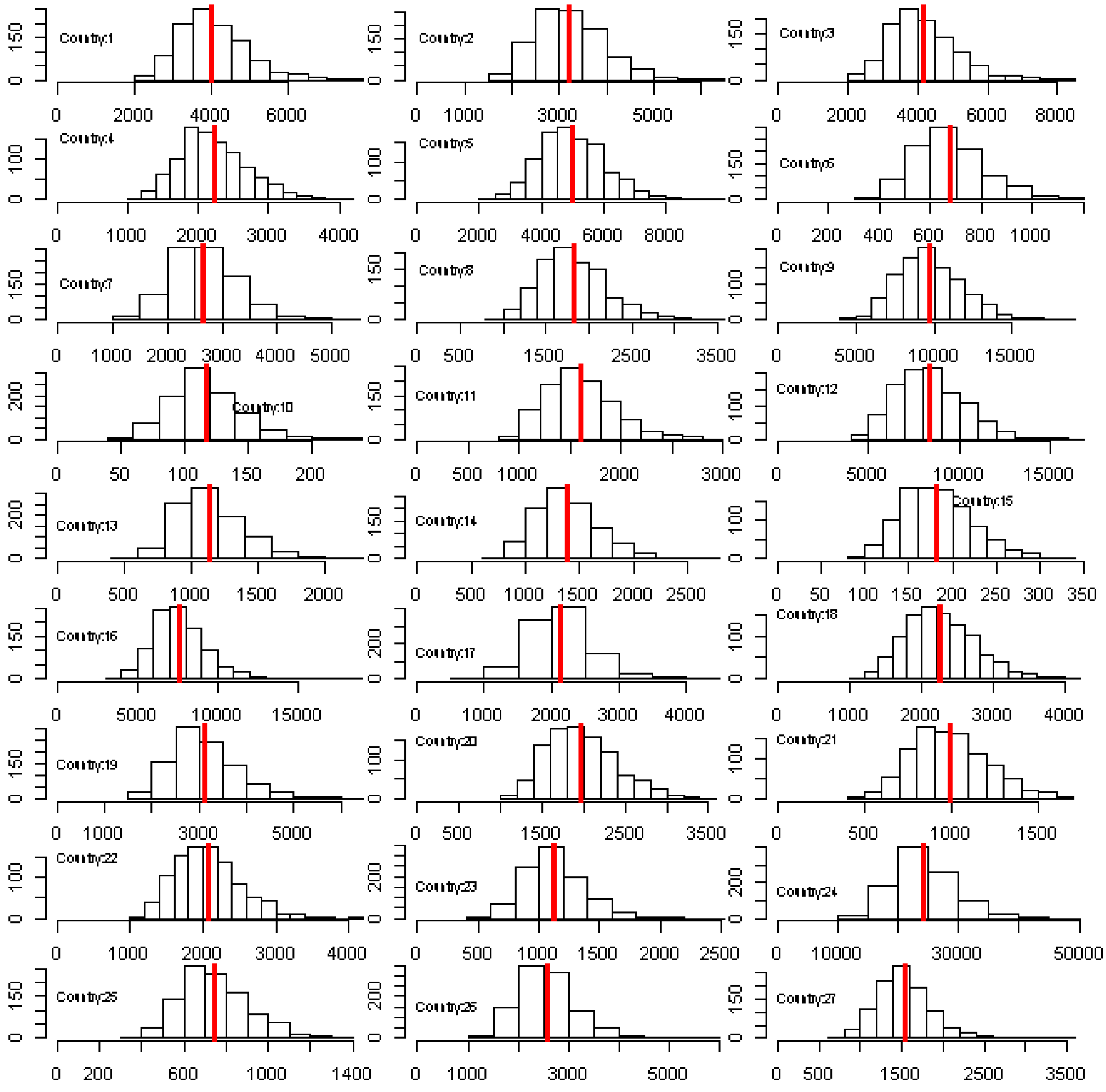

Web Figure 3. Distribution of the simulated cases (histogram based on C_i^j j=1,…,1000) for entity 3 (in red: actual observed cases in the corresponding country, that is C_i) for each of the 27 countries considered.

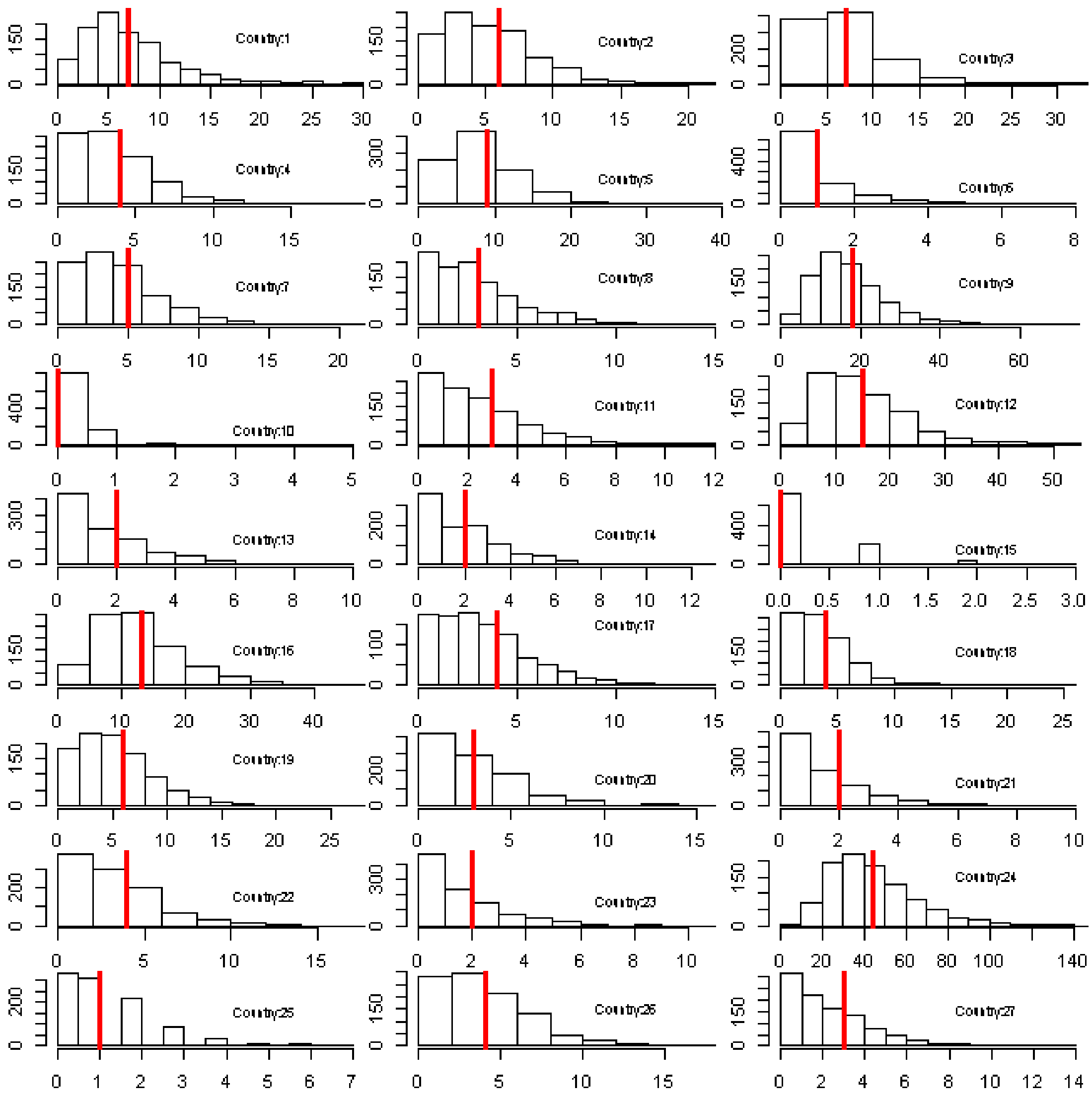

Web Figure 4. Distribution of the simulated cases (histogram based on C_i^j j=1,…,1000) for entity 4 (in red: actual observed cases in the corresponding country, that is C_i) for each of the 27 countries considered.

WEB APPENDIX 3

Procedures and Models Used in the Simulation Study

(I) Exact confidence intervals for λ_i

We first proceed with the simplest approach based on the exact Poisson approximation to the confidence limits since this approach performs well. We used epitools R library (see its description in http://www.epitools.net, last accessed November 2019), which allows for computing these confidence intervals, and we referred to them as *'exact Poisson'* in the tables.

(II) Byar's approximation to confidence interval for λ_i

Another one of the simplest approaches, based on assuming a non-random-effects model from the classical perspective, is to use the common Byar's approximation to $100(1-\alpha)\%$ confidence interval based on Poisson counts (1, 2)

$$\lambda_{L_i}^j = C_i^j(1 - \frac{1}{9\,C_i^j} - \frac{Z_{\frac{\alpha}{2}}}{3\left(C_i^j\right)^{0.5}})^3$$

(E1),

$$\lambda_{U_i}^j = (C_i^j + 1)(1 - \frac{1}{9(C_i^j + 1)} - \frac{Z_{\frac{\alpha}{2}}}{3\left(C_i^j + 1\right)^{0.5}})^3$$

where λ_{L_i} and λ_{U_i} are the respective lower and upper bounds for the confidence limits, and $Z_{\frac{\alpha}{2}}$ denotes the $100\left(1 - {}^{\alpha}/_{2}\right)$ percentile of the standard normal distribution.. We used epitools R library, which allows for computing these confidence intervals, and we referred to them as '*Byar Poisson'* in the tables.

(III) Empirical Bayes approach by assuming Gamma distribution for R_i^j

Two random-effects models were assessed using the maximum likelihood approach. First, we followed the EB proposal by Clayton and Kaldor (3) based on the assumption that R_i^j follows a common gamma distribution with scale parameter β and shape parameter α parameterized as

$$f(R_i^j) = \frac{\beta^\alpha (R_i^j)^{\alpha-1} e^{-\beta R_i^j}}{\Gamma(\alpha)}. \qquad \text{(E2)}$$

Then the distribution of R_i^j conditional on C_i^j is also Gamma with expectation $(C_i^j + \alpha)/(E_i + \beta)$. Simulating from this distribution can yiedld the required quantiles for the R_i^j. Moreover, the unconditional expectation of C_i^j is $E_i \frac{\alpha}{\beta}$ since C_i^j follows a negative binomial distribution (3). In this line, maximum likelihood estimates for (α, β) can be obtained through a generalized linear model assuming a negative binomial for C_i^j as described by McCullagh and Nelder (4). This approach is implemented in the R library SpatialEpi (5) and the model is referred to as EB in the tables.

(IV) Generalized linear mixed model

The performance of the model (M1)

$$\theta_i = \log(R_i) = \mu + \nu_i \, , i = 1, \dots, I = 27, \quad \text{(M1)}$$

$$\nu_i \sim N(0, \tau),$$

was also evaluated under the maximum likelihood approach by incorporating both fixed-effect parameters, μ, and random effects, ν_i, in the linear predictor through a generalized linear mixed model. We used the glmer function of the lme4 package in R (6), whereas the quantiles of the distribution of the fitted R_i^j, and therefore, C_i^j values were obtained through simulation using the R package arm. This model is referred to as GLM-ML in the tables.

(V) Prior precision for the models estimated using INLA and WinBUGS

Two prior distributions for τ were used to estimate (M1) using INLA: the first prior distribution used was a Gamma distribution (INLA's default) with the same parametrization used in equation E2, properly adapted for τ instead of R_i^j , with parameters $\alpha = \beta =$ 0.00001; for the second one we assumed an approximation to $\sigma \sim Uniform(0, \infty)$. Since INLA uses an internal representation of the parameters working with $\theta = \log(\tau)$, this prior must be specified on θ, therefore, since $\pi(\theta) = \pi(\sigma) |\frac{\partial\sigma}{\partial\theta}|$ then $\pi(\theta) = \pi(\sigma) \left|\frac{1}{2}\exp(-\frac{\theta}{2})\right|$. See Krainski et al (7) for more

details. The uniform prior on σ must be a uniform between 0 and infinity (for computational reasons), therefore changing lower bounds for Uniform can't be assessed, to date, using INLA (see Gómez-Rubio et al for details (8) and its R-code implementation in Web Appendix 4).

In the same line, there were several WinBUGS assessed: There were 7 WinBUGS models fitted by assuming that σ follows a Uniform prior with different LB and UB (see their code implementation in section 1): M1) LB=0 and UB=500; M2) LB=0.1 and UB=500; M3) LB= 0.2 and UB=500; M4) LB=0.3 and UB=500; M5) LB=0.5 and UB=500; M6) LB=1 and UB=500; and M7) by sampling from LB and UB such that LB$\sim Uniform(0,5)$ and UB$\sim Uniform(LB, 500)$. See Web Appendices 1 and 5 for R-code implementation. Note that INLA models are referred here as M8 and M9.

WEB APPENDIX 4

Integrated Nested Laplace Approximation (INLA) Code When Assuming $\sigma \sim \mathbf{Uniform}(0, \infty)$

```
#INLA Prior SD

UN.prior = ‘expression:

  log_dens = 0 - log(2) - theta / 2;

  return(log_dens);

‘

unprior = list(prec = list(prior = UN.prior))

# Model

#Observed: Observed Cases

#Country: Country code from i=1,…,27

#ExpectedEU: Expected cases according to the European distribution of counts

mod.INLA.SD<-
inla(Observed~f(factor(Country),model=‘iid’,hyper=unprior),family=‘poisson’,E=ExpectedEU
,data=datos.temp,control.predictor=list(compute=TRUE,link=1),control.compute=list(dic=T,
mlik=T,config=T),control.results=list(return.marginals.predictor=T),verbose=F)
```

WEB APPENDIX 5

Winbugs Code: Poisson Random-Effects Model Sampling LB and UB

```
for (i in 1:n.countries)
{
              C[i] ~ dpois(lambda[i])
              log(lambda[i]) <- log(E[i]) + mu + nu[i]
              nu[i] ~ dnorm(0, tau) # area random effects
              theta[i] <- mu + nu[i] # area log-relative risk
              R_hat[i]<-exp(theta[i])
              # Posterior expectancy of Cases based on the R[i] posterior
              C_hat[i]<-R_hat[i]*E[i]
}

mu ~ dflat() # uniform prior on overall intercept
LB~dunif(0,5)
UB~dunif(LB,500)
sigma ~ dunif(LB, UB) # sd area-random effects
tau <- 1/pow(sigma,2) # between-area precision
```

WEB APPENDIX 6

Measures for the Assessment of the Models/Methods in the Simulation Study

For each simulated data set, the average width of the confidence – procedures I,II,III – or credible – procedure IV – intervals was calculated across the I=27 countries, as well as the percentage of countries for which the 'real' expected count, $R_i^j E_i, i = 1, \dots, 27$ was included into the corresponding 95% confidence/credible interval, the coverage, as well as the root of the mean square error (RMSE) of $\sum_{i=1}^{27} \frac{\left(\lambda_i^j - \hat{\lambda}_i^j\right)^2}{27}$. For each cancer entity, tables of results for these indicators present their overall mean, their minimum and maximum values, all obtained across the j=1,…,1000 simulated data sets.

In addition to these indicators, in the full Bayesian setting we compared the goodness-of-fit based on the DIC (calculated through combining the effective number of model parameters, pD) and the mean deviance, $\overline{D(R^j)}$, where $\overline{R^{\,j}} = \{R_1^j, \dots, R_{27}^j\}$. These indicators were calculated as follows

$$D(R^j) = -2\sum_i \log(p(C_i^j | R^j, E_i)),$$

$$pD_{WinBUGS} = \overline{D(R^j)} - D(R^j),$$

$$pD_{INLA} = I - tr\{Q(R^j)Q^*(R^j)^{-1}\},$$

$$DIC = D(R^j) + pD.$$

In WinBUGS, the estimate of the average of $D(R^j)$ over a sample from the converged Markov chain leads to $\overline{D(R^j)}$ (7-11). However, in INLA, obtaining $\overline{D(R^j)}$ through $D(R^j)$ requires two steps(7,8): i) computing the conditional mean using univariate numerical integration for each i=1,..,I and ii) integrating R^j out of the expression with respect to $p(R^j | C^j)$. Then, unlike with WinBUGS, the deviance for INLA is calculated at the posterior mean/mode of the latent field rather than the posterior mean of all parameters. The indicator $pD_{WinBUGS}$ is the classical definition of pD (9,10) and $D(R^j)$ is calculated as the deviance computed at the posterior mean estimates(8-10). Calculating pD in INLA requires I, the number of observations (countries in our study), and two matrices, that of the prior precision $Q(R^j)$ and that of the posterior covariance matrix of the Gaussian approximation $Q^*(R^j)$ (7,8,11). We kept these estimates over the 1000 simulated data sets. However, a lower value in one of these platforms does not necessarily mean that one platform fits better than the other, as the DIC calculations differ

between platforms. In this situation, the coverage and mean squared error indicators described in the main text might be better for comparing across platforms(12-14).

WEB APPENDIX 7

Computational Issues in the Simulation Study

The INLA approach to approximate the posterior distribution of the hyperparameters can be changed to increase accuracy or reduce computation time (8, 11, 12). In this line, the uniform prior on σ must be a uniform between 0 and ∞ (for computational reasons), so changing lower bounds for Uniform cannot currently be assessed using INLA (8). Results presented here are based on the initial setting in INLA. We used the stable version of INLA (available at http://www.r-inla.org/download), downloaded 10 March 2018, and WinBUGS version 1.4.3, downloaded on 10 March 2018 at the *mrc repository* (https://www.mrc-bsu.cam.ac.uk/software/). R2WinBUGS(10) was the library used to run WinBUGS in R. In order to obtain comparable results, we ran WinBUGS models with three Markov chains (length 4,000). We checked the Gelman-Rubin diagnostic estimate of the potential reduction scale (12-14) to assess convergence of the Markov chains.

WEB APPENDIX 8

WAIC and Log Predictive Pointwise Density (LPPD) Estimation Using Markov Chain Monte Carlo (MCMC)

Since our aim was to estimate predictive accuracy of M1,..,M8, we used the LPPD (13-17) and from there, calculated the WAIC(15,16) through MCMC for model comparison purposes and as a measure of model accuracy. LPPD can be computed based on the estimates derived from MCMC (17):

$$LPPD_{MCMC} = \sum_{i=1}^{I} log\left(\frac{1}{S}\sum_{s=1}^{S} p(C_i|R_i^s, E_i)\right) \qquad \text{(E3)},$$

where p is a Poisson distribution, S is the number of simulation draws and R_i^s is the s-th simulated value from the posterior distribution of R_i. From (E3), one can derive an estimate of the WAIC (17), a fully Bayesian approach for estimating the out-of-sample expectation by using (E3) and adding a correction for the effective number of parameters to adjust for overfitting, the p_{WAIC}, leading to two WAIC estimates, WAIC1 and WAIC2 (13-17). This can be calculated two ways, leading to two WAIC estimates (17) as follows:

$$WAIC_t = -2\left(LPPD_{MCMC} - p_{WAIC_t}\right), t = \{1,2\},$$

$$p_{WAIC_1} = 2\sum_{i=1}^{I}[log\left(\frac{1}{S}\sum_{s=1}^{S} p(C_i|R_i^s, E_i)\right) - \frac{1}{S}\sum_{s=1}^{S} p(C_i|R_i^s, E_i)],$$

$$p_{WAIC_2} = \sum_{i=1}^{I} Var_{s=1}^{S}(p(C_i|R_i^s, E_i)).$$

Although Gelman recommended the use of $WAIC_2$ because its series expansion resembles the series expansion of the leave-one-out cross validation (17), we assessed both $WAIC_1$ and $WAIC_2$. A note on the model selection comparing WAIC with other indicators as well as it implementation in R can be found in Web Appendices 9 and 10, respectively.

WEB APPENDIX 9

A Note on Model Selection Methods Used in this Paper

Model selection requires a compromise between goodness-of-fit and flexibility, quantifying these two criteria through some metric. The four frequently used methods implementing information criteria are: the Akaike information criterion (AIC) (18), the Bayesian information criterion (BIC) (19), the deviance information criterion (DIC) (20), and the widely applicable information criterion (WAIC) (15,16). DIC and WAIC estimates can be obtained from INLA, but WAIC is not available from a WinBUGS output.. Web Appendix 10 shows its R code implementation when using MCMC samples derived from WinBUGS.

AIC and BIC metrics require simple frequentist methods for parameter estimation, using the maximum likelihood estimate as the measure of goodness-of-fit, plus a transformation of the number of free (‘effective’) parameters in the model as a measure of the flexibility. However, this last counting of parameters cannot answer to how the selected model’s function could modify the flexibility provided by each parameter (21). On the other hand, DIC and WAIC both demand Bayesian parameter estimation with (i) goodness-of-fit calculated as the average likelihood of the data given the parameters – that is $p(y|\theta)$) over the entire posterior- and (ii) flexibility calculated over a measure of variability of the likelihood’s posterior (17,20).

DIC and WAIC aim to provide an approximation of predictive accuracy, and they are asymptotically equivalent to leave-one-out cross validation (LOO-CV) (17). DIC uses the difference between the average of the log-likelihood over the posterior distribution (as a measure of goodness-of-fit) and the log-likelihood at a central point of the posterior as a measure of flexibility, that is the effective number of model parameters (*pD)*. Greater differences resulting in model penalization: WAIC uses log predictive pointwise density (LPPD), a similar measure of goodness-of-fit to DIC. LPPD is the log of the average posterior likelihood for each data point, but WAIC also uses the variance in log-likelihood over the posterior distribution as a measure of flexibility, p_{WAIC}, with greater variances resulting in model penalization. Since DIC can be expressed as a function of pD and WAIC as a function of p_{WAIC}, analysts selecting models using these two criteria are interested in choosing the model with minimum DIC and minimum WAIC. Since selecting a model using only one of these indicators can lead to different choices (21), we have suggested selecting the Bayesian models according to a ranking derived from WAIC and DIC indicators. The procedure to derive this models’ ranking is illustrated in Web Appendix 11.

WEB APPENDIX 10

R Code: WAIC Calculation From WinBUGS Output

```
Likelihood.single.P = function(y, theta, Expect.t,pos.vec) {

  #Get the individual parameters from theta.

 # theta: vector of samples; pos.vec: position into vector y,Expect.t

dpois(y[pos.vec], theta[,pos.vec]*Expect.t[pos.vec])

}

calculate_WAIC.P = function(y, theta,Expect.t, likeFun) {

    # Set up variables

    LPPD = 0

    P_1 = 0

    P_2 = 0

        for (i in 1:length(y)) {

        # Get a vector of likelihoods for this observation in y

           L = likeFun(y, theta,Expect.t,i)

        # Calculate LPPD for this observation

        LPPD = LPPD + log( mean(L) )

        # Calculate P_1

        a = log( mean(L) )

        b = mean( log(L) )

        P_1 = P_1 + 2 * (a - b)

        # Calculate P_2

        P_2 = P_2 + var( log(L) )

    }

    # WACI two ways, they differ through P

    WAIC_1 = -2 * (LPPD - P_1)

    WAIC_2 = -2 * (LPPD - P_2)

    # Return a list containing the results

    list(WAIC_1 = WAIC_1, WAIC_2 = WAIC_2, P_1 = P_1, P_2 = P_2, LPPD = LPPD)

}

# USE: Observed and Expected are vectors of N=27 counts, the example uses Model1.txt

WinBUGS.model <-
bugs(data,inits,model.file='C:/Study/Model1.txt',parameters=c('lambda','tau','alpha'),n.
chains=3,n.iter=20000,n.burnin=10000,n.thin=10,bugs.directory='c:/WB',codaPkg=FALSE,debu
g=F)

calculate_WAIC.P(y=Observed,theta=WinBUGS.model$sims.matrix[,1:27],Expect.t=Expected,lik
eFun=Likelihood.single.P)
```

WEB APPENDIX 11

Illustration on the Calculation of Models' Ranking

The Web Figure 5 depicts how to rank models for each entity. Assume that we have 190 entities and 7 models. The indicators are stored in a matrix M of 190 rows (entities) and 8(models) × 5(indicators), giving 40 columns. The procedure is as follows: for each cancer entity the model rank was calculated according to each of the indicators (see Step 2), and this generated the matrix of rankings R. Averaging the rankings of indicators for each model in the matrix R, the 'ranking' for each model and cancer site could be determined.

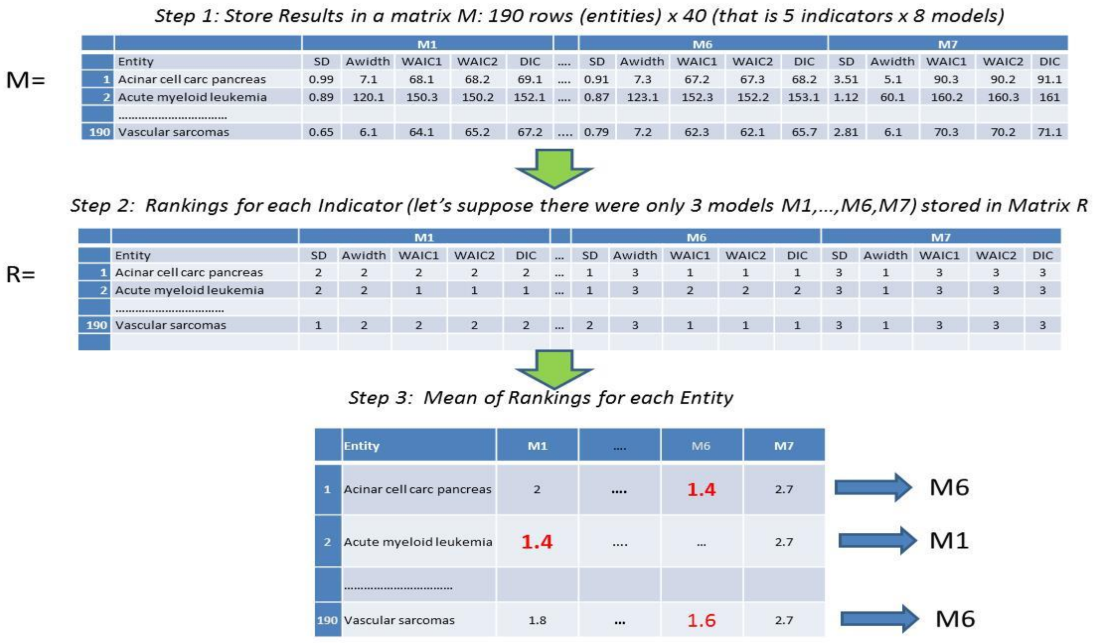

		M1						M6					M7				
	Entity	SD	Awidth	WAIC1	WAIC2	DIC	....	SD	Awidth	WAIC1	WAIC2	DIC	SD	Awidth	WAIC1	WAIC2	DIC
1	Acinar cell carc pancreas	0.99	7.1	68.1	68.2	69.1	....	0.91	7.3	67.2	67.3	68.2	3.51	5.1	90.3	90.2	91.1
2	Acute myeloid leukemia	0.89	120.1	150.3	150.2	152.1	....	0.87	123.1	152.3	152.2	153.1	1.12	60.1	160.2	160.3	161
	....																
190	Vascular sarcomas	0.65	6.1	64.1	65.2	67.2	....	0.79	7.2	62.3	62.1	65.7	2.81	6.1	70.3	70.2	71.1

		M1						M6					M7				
	Entity	SD	Awidth	WAIC1	WAIC2	DIC	...	SD	Awidth	WAIC1	WAIC2	DIC	SD	Awidth	WAIC1	WAIC2	DIC
1	Acinar cell carc pancreas	2	2	2	2	2	...	1	3	1	1	1	3	1	3	3	3
2	Acute myeloid leukemia	2	2	1	1	1	...	1	3	2	2	2	3	1	3	3	3
	....																
190	Vascular sarcomas	1	2	2	2	2	...	2	3	1	1	1	3	1	3	3	3

	Entity	M1	....	M6	M7
1	Acinar cell carc pancreas	2	....	1.4	2.7
2	Acute myeloid leukemia	1.4	....	...	2.7
	....				
190	Vascular sarcomas	1.8	...	1.6	2.7

Web Figure 5. Steps to rank models for each entity.

In this line, for deriving model rankings for each scenario, cancer entities must be selected according to the scenario's criterion, the IRs. Therefore, one must select the corresponding rows in the matrix R. This is a new matrix for the corresponding scenario, say R-SC. Then, the average of each models' ranking in R-SC must be calculated.

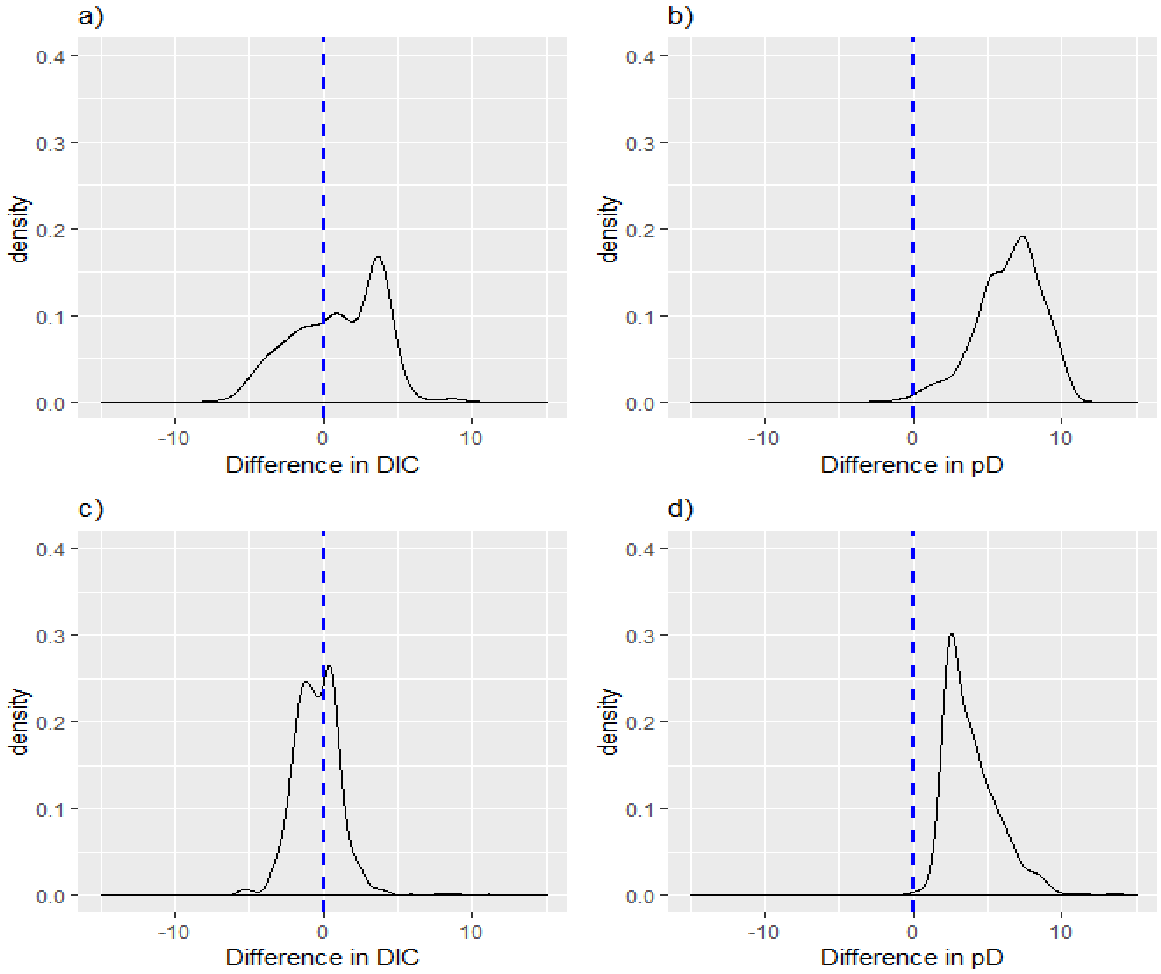

Web Figure 6. Differences in DIC and pD between INLA models M8 (Gamma prior on τ) and M9 (Uniform prior on σ) across J=1000 simulated data sets: Panels a) and b) "adenocarcinoma with variants of middle ear", Panels c) and d) "adenocarcinoma with variants of trachea."

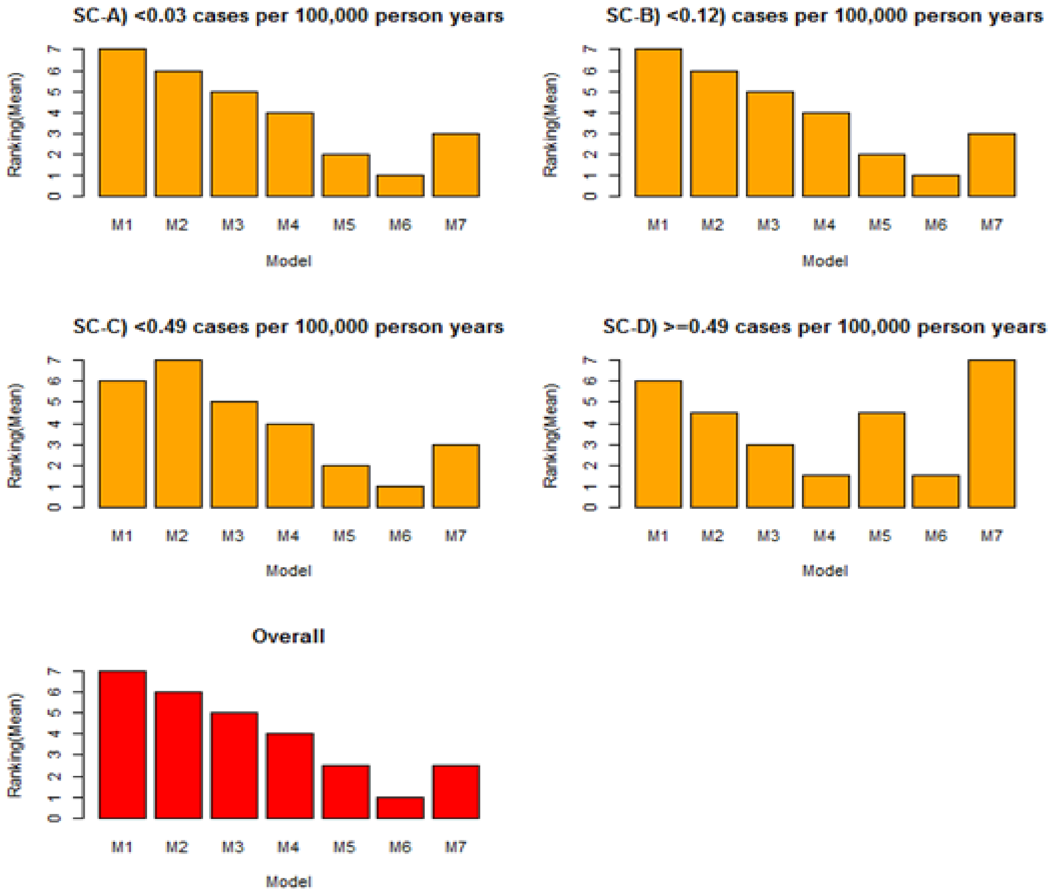

Web Figure 7. Ranking of models accounting for DIC, WAIC1 and WAIC2, stratified by scenarios as well as overall ranking (non-stratified by scenarios). Overall ranking was calculated by averaging DIC and WAIC rankings into one indicator.

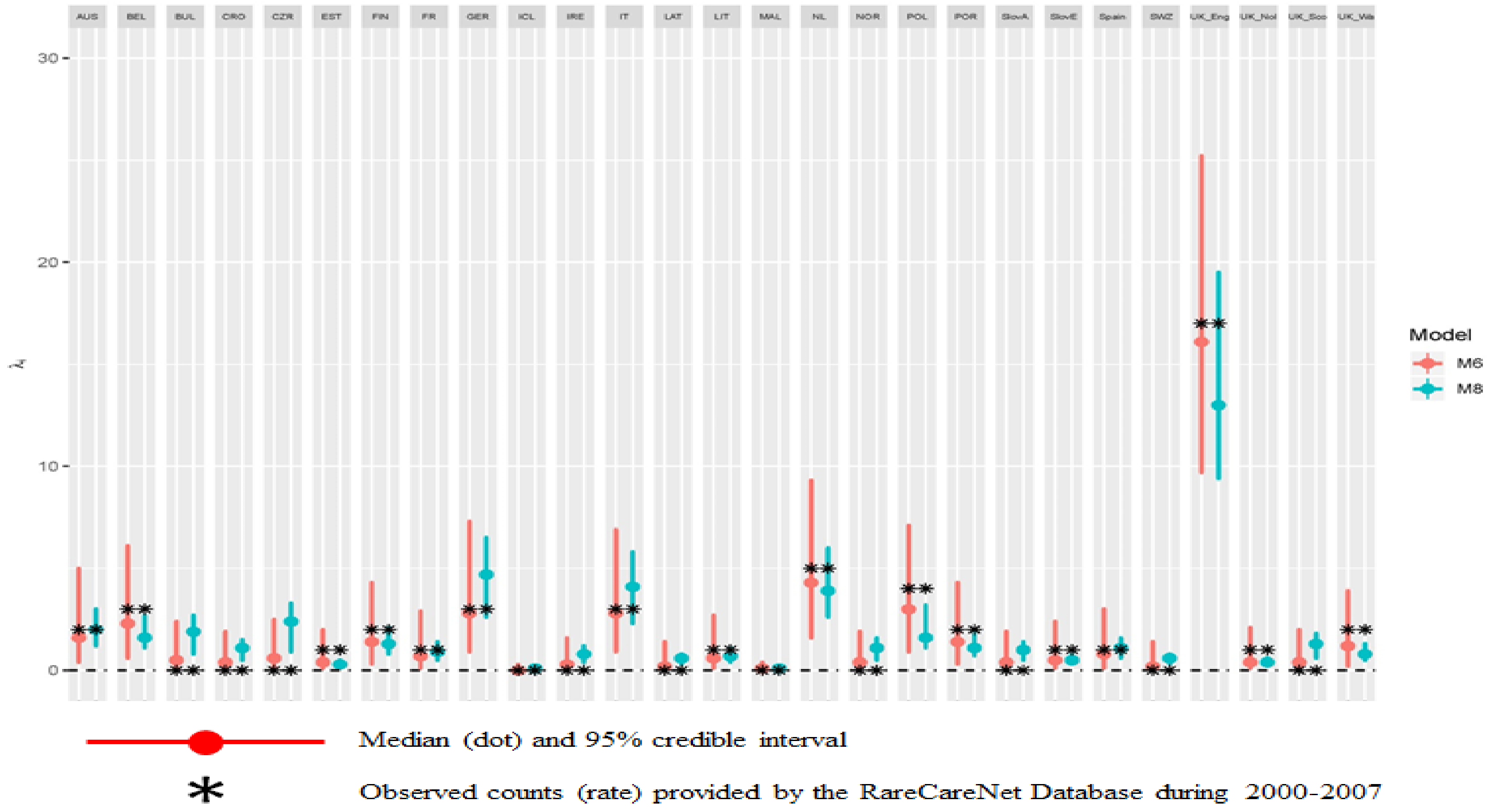

Web Figure 8. Comparison of the 95% credible interval coverage obtained through model M6 and model M8 for adenocarcinoma with variants of middle ear across countries considered in RareCareNet.

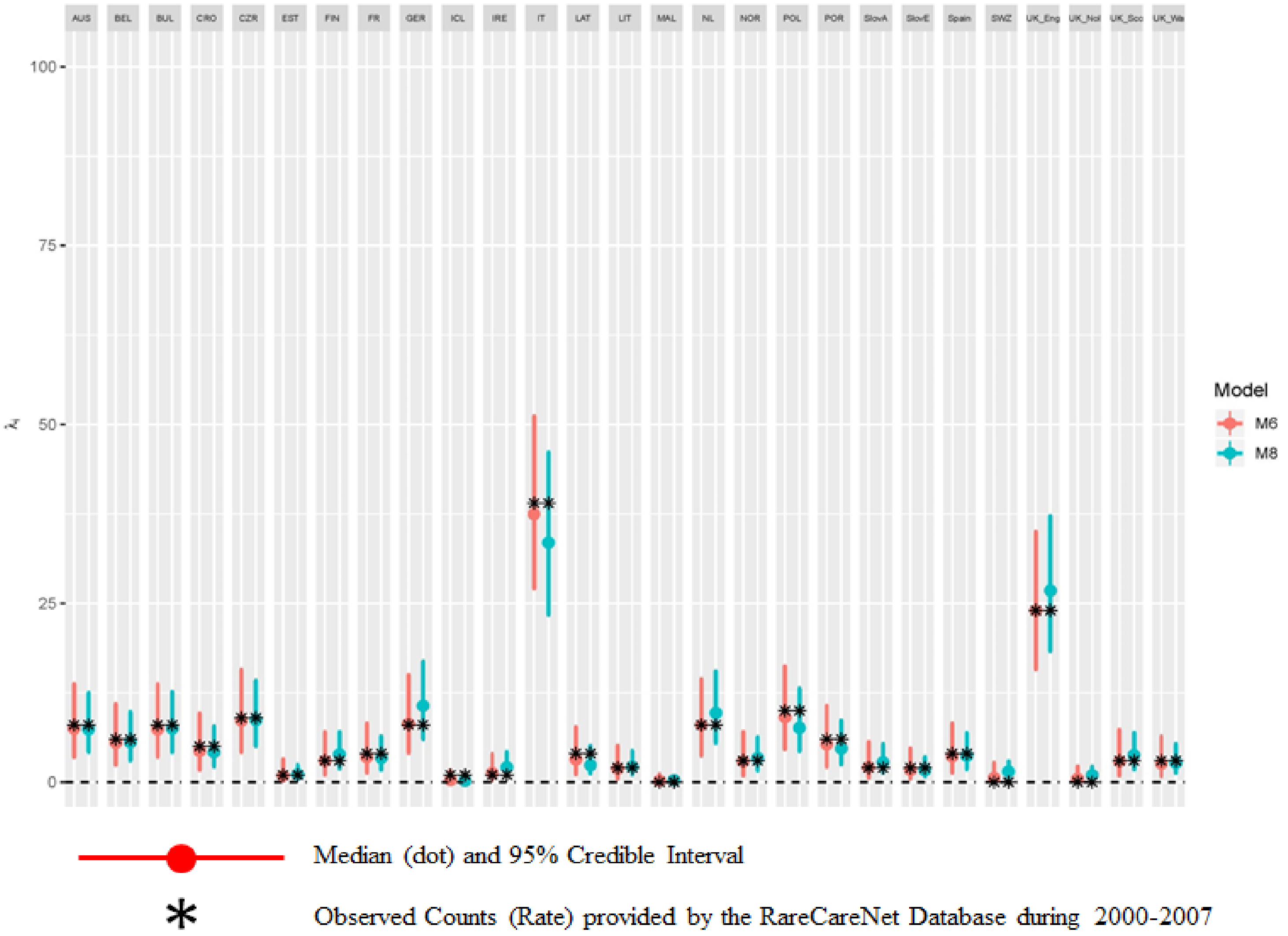

Web Figure 9. Comparison of the 95% credible interval coverage obtained through model M6 and model M8 for adenocarcinoma with variants of trachea accross countries considered in RareCareNet.

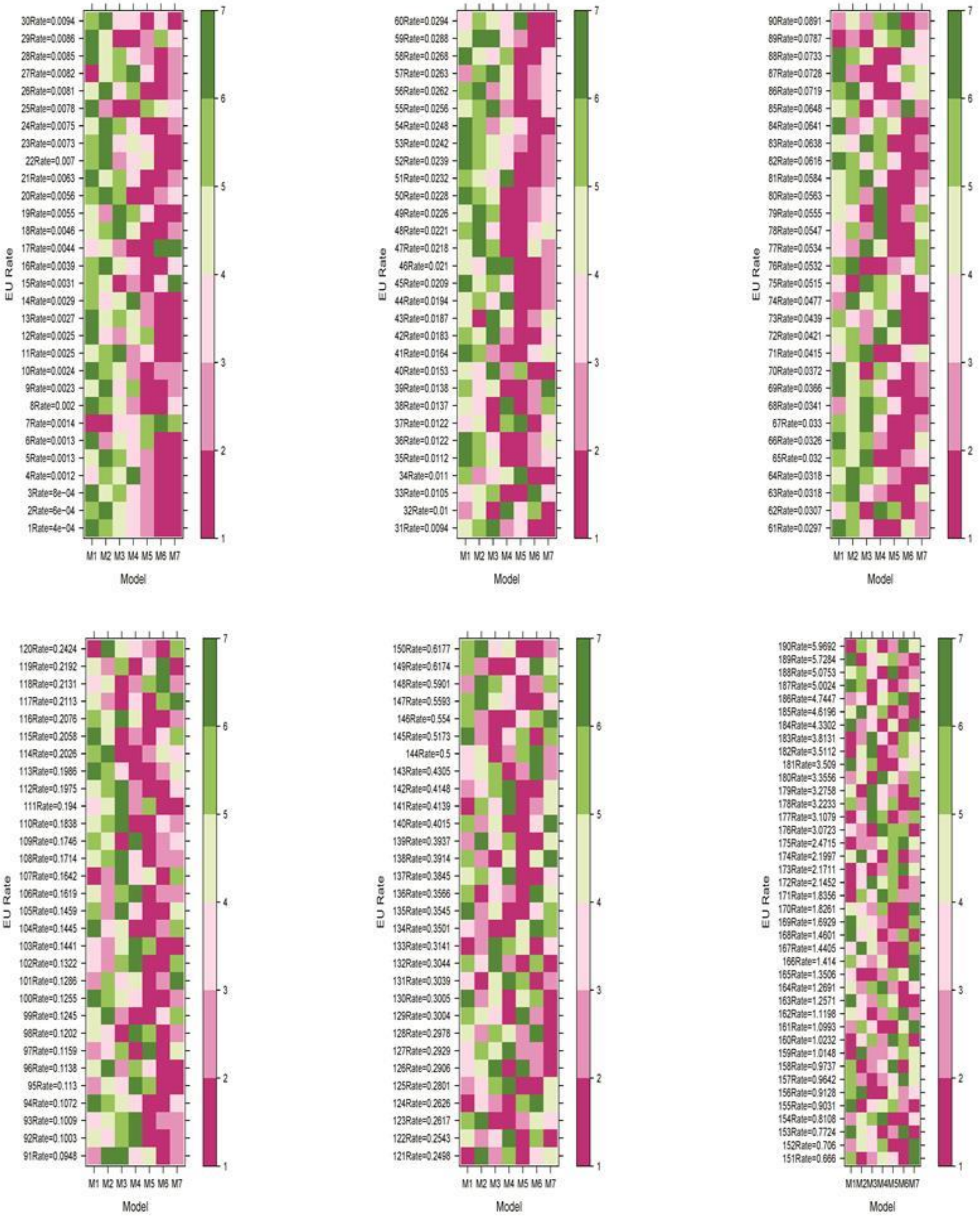

Web Figure 10. Ranking of models across the 190 cancer entities considered. Entities ordered according to the mean number of cases.

Web References

1. Swift, B. Comparison of confidence intervals for a Poisson mean – further considerations. *Comm in Stat—Theory and Methods.* 2009. 38: 748–759.

2. Browne WJ and Draper D. A comparison of Bayesian and likelihood-based methods for fitting multilevel models. *Bayesian Analysis*. 2006. 1:473-514.

3. Clayton D, Kaldor J. Empirical Bayes estimates of age-standardized relative risks for use in disease mapping. *Biometrics*. 1987. 43:671–81.

4. McCullagh P, Nelder JA. *Generalized Linear Models*. (Monographs on Statistics and Applied Probability, Vol. 37). 2nd ed. London: Chapman and Hall; 1989.

5. Kim AY, Wakefield J. SpatialEpi: Methods and Data for Spatial Epidemiology. R package version 1.2.3 (2018). url: https://CRAN.R-project.org/package=SpatialEpi.

6. Bates D., Mächler M., Bolker B., Walker, S. Fitting Linear Mixed-Effects Models Using lme4. *Journal of Statistical Software*. 2015: 67(1), 1–48.

7. Krainski ET, Gómez-Rubio V, Bakka H et al. *Advanced Spatial Modeling with Stochastic Partial Differential Equations Using R and INLA*. London: Chapman and Hall; 2019.

8. Gómez-Rubio V, Palmí-Perales F, López-Abente G et al. Bayesian joint spatio-temporal analysis of multiple diseases. *SORT*. 2019. 43(1):1-24.

9. Lunn, D, Jackson C, Best N, et al. *The BUGS Book: A Practical Introduction to Bayesian Analysis*. Boca Raton, FL: CRC Press; 2003.

10. Sturtz, S., Ligges, U., Gelman, A. R2WinBUGS: A Package for Running WinBUGS from R. *Journal of Statistical Software*. 2005. 12(3), 1-16.

11. Rue H, Martino S, Chopin N. Approximate Bayesian inference for latent Gaussian models by using integrated nested Laplace approximations. *J Royal Stat Soc B*. 2009. 71: 319–392.

12. Carroll R, Lawson AB, Faes C, et al. Comparing INLA and OpenBUGS for hierarchical Poisson modeling in disease mapping. *Spat Spatiotemporal Epidemiol*. 2015 Jul-Oct;14-15:45-54.

13. Gelman, A, Rubin DB. Inference from iterative simulation using multiple sequences. *Stat Sci*. 1992. 7: 457–511.

14. Gelman A, Hwang J, Vehtari A Understanding predictive information criteria for Bayesian models. *Statistics and Computing.* 2016. 24(6):997-1016.

15. Watanabe S. Asymptotic equivalence of Bayes cross validation and widely applicable information criterion in singular learning theory. *J Machine Learning Res*. 2010. 11: 3571–3594.

16. Watanabe S. A widely applicable Bayesian information criterion. *J Machine Learning Res*. 2013. 14: 867–897.

17. Vehtari, A., Gelman, A., & Gabry, J. (2017). Practical Bayesian model evaluation using leave-one out cross-validation and WAIC. *Statistics and Computing*, 27(5), 1413–1432.

18. Akaike, H. (1974). A new look at the statistical model identification. *IEEE Transactions on Automatic Control*, 19(6), 716–723.

19. Schwarz, G. (1978). Estimating the dimension of a model. *The Annals of Statistics*, 6(2), 461–464.

20. Spiegelhalter, D.J., Best, N.G., Carlin, B.P., & Van Der Linde, A. (2002). Bayesian measures of model complexity and fit. *Journal of the Royal Statistical Society: Series B (Statistical Methodology)*, 64(4), 583–639.

21. Myung, I.J. (2000). The importance of complexity in model selection. *Journal of Mathematical Psychology*, 44 (1), 190–204.
